# Competitive control of endoglucanase gene *engXCA* expression in the plant pathogen *Xanthomonas campestris* by the global transcriptional regulators HpaR1 and Clp

**DOI:** 10.1111/mpp.12739

**Published:** 2018-10-09

**Authors:** Guo‐Fang Liu, Hui‐Zhao Su, Han‐Yang Sun, Guang‐Tao Lu, Ji‐Liang Tang

**Affiliations:** ^1^ State Key Laboratory for Conservation and Utilization of Subtropical Agro‐bioresources, College of Life Science and Technology Guangxi University 100 Daxue Road Nanning Guangxi 530004 China

**Keywords:** competitive activation, co‐regulate, overlapping sites, transcriptional regulator, *Xanthomonas*.

## Abstract

Transcriptional regulators are key players in pathways that allow bacteria to alter gene expression in response to environmental conditions. However, work to understand how such transcriptional regulatory networks interact in bacterial plant pathogens is limited. Here, in the phytopathogen *Xanthomonas campestris*, we demonstrate that the global transcriptional regulator HpaR1 influences many of the same genes as another global regulator Clp, including the *engXCA* gene that encodes extracellular endoglucanase. We demonstrate that HpaR1 facilitates the binding of RNA polymerase to the *engXCA* promoter. In addition, we show that HpaR1 binds directly to the *engXCA* promoter. Furthermore, our *in vitro* tests characterize two binding sites for Clp within the *engXCA* promoter. Interestingly, one of these sites overlaps with the HpaR1 binding site. Mobility shift assays reveal that HpaR1 has greater affinity for binding to the *engXCA* promoter. This observation is supported by promoter activity assays, which show that the *engXCA *expression level is lower when both HpaR1 and Clp are present together, rather than alone. The data also reveal that HpaR1 and Clp activate *engXCA* gene expression by binding directly to its promoter. This transcriptional activation is modulated as both regulators compete to bind to overlapping sites on the *engXCA* promoter. Bioinformatics analysis suggests that this mechanism may be used broadly in *Xanthomonas campestris* pv. *campestris* (*Xcc*) and is probably widespread in Xanthomonads and, potentially, other bacteria. Taken together, these data support a novel mechanism of competitive activation by two global regulators of virulence gene expression in *Xcc* which is probably widespread in Xanthomonads and, potentially, other bacteria.

## Introduction

Bacterial pathogens continuously adjust their gene expression in response to the environment in order to control and deploy appropriate virulence agents. Transcription factors are one way in which bacteria can achieve control over gene expression. These proteins can sense signals and specifically activate or inhibit target genes that are involved in physiological adaptation, pathogenesis and virulence. In general, transcriptional regulator proteins consist of an N‐terminal domain which bears a helix–turn–helix (HTH) DNA‐binding motif and a C‐terminal regulatory domain which signals bind. These factors are also classed as ‘activators’ when they bind to a promoter to activate transcription initiation, and/or ‘repressors’ when they bind to a promoter to interrupt transcription (Browning and Busby, 2016; Ishihama, 2010). Although transcription factors can function solely as activators or repressors, there are examples of these proteins functioning according to where they bind to the target promoter (Browning and Busby, 2016; Ishihama, 2010). Generally, repressors bind downstream of promoters, thus blocking the recognition of RNA polymerase (RNAP) holoenzymes to promoters. Conversely, activators bind upstream of promoters, driving the recruitment of RNAP to the target promoters.

Bacteria belonging to the genus *Xanthomonas* cause diseases in many economically important plants, leading to extensive decreases in crop yields and quality worldwide (Swings and Civerolo, 1993; Vicente and Holub, 2013). These phytopathogens use a wide range of transcription factors to regulate the expression of gene encoding factors contributing to virulence (Ryan *et al.*, 2011, 2015; Zhou *et al.*, 2017). The paradigm for many of these factors deployed by *Xanthomonas *strains has been described from studies using the cruciferous pathogen *Xanthomonas campestris* pv. *campestris* (*Xcc*), a model bacterium used over the past several decades to study plant–pathogen interactions (Ryan *et al.*, 2011, 2015; Zhou *et al.*, 2017). In *Xcc*, several global transcriptional regulators have been described to play a major role in pathogenesis, including Clp (CRP‐like protein), Zur (zinc uptake regulator), HpaR (for *hrp*‐associated regulator) and HpaR1 (for *hrp*‐associated regulator) (An *et al.*, 2011; Chin *et al.*, 2010; de Crecy‐Lagard *et al.*, 1990; Huang *et al.*, 2009; Su *et al.*, 2016; Tang *et al.*, 2005; Wei *et al.*, 2007). Clp has been shown to positively regulate the synthesis of extracellular polysaccharide (EPS), extracellular enzymes and virulence, and its action is governed by the binding of the intracellular second messenger cyclic diguanylate (cyclic‐di‐GMP) (Chin *et al.*, 2010; de Crecy‐Lagard *et al.*, 1990). HpaR, which encodes a putative MarR family regulator, has been demonstrated to regulate virulence, the hypersensitive response and extracellular protease production (Wei *et al.*, 2007). Moreover, recent studies have described how Zur, a member of the Fur family of transcriptional regulators, is a key element in the control of zinc homeostasis and positive regulation of the type III secretion system (T3SS) (Huang *et al.*, 2009; Tang *et al.*, 2005), More recently, our work has demonstrated how the HpaR1 protein, a global regulator belonging to the YtrA subfamily of the GntR family, regulates virulence, EPS production, extracellular enzymes and T3SS during *Xcc* infection (An *et al.*, 2011; Su *et al.*, 2016).

The global transcriptional regulators characterized in *Xcc* illustrate the complex network of regulation they control and the pleiotropic phenotypes modulated by their action. Despite studies to understand how such bacterial transcriptional regulatory networks work, only limited information is available on how they functionally interact. Here, we demonstrate that the global transcriptional regulator HpaR1 influences many of the same genes as another global regulator Clp. Although both transcriptional regulators Clp and HpaR1 have been shown to control the activity of extracellular enzymes in *Xcc* (de Crecy‐Lagard *et al.*, 1990; Su *et al.*, 2016), no molecular analysis of the role of HpaR1 in the expression of extracellular enzyme encoding genes has been reported. Accordingly, our efforts aimed to elucidate the HpaR1 regulation of the expression of the major endoglucanase encoding gene *engXCA* at a molecular level, and to understand the interplay of regulation with Clp. We show that HpaR1 binds directly to the promoter of *engXCA* to enhance its expression, and that the specific promoter binding site has been identified and characterized using site‐directed mutagenesis. The interplay between HpaR1 and Clp on the expression of *engXCA *was also revealed using electrophoretic mobility shift assay (EMSA) and *in vitro *transcriptional analysis. This analysis showed that the binding site of HpaR1 to the *engXCA* promoter overlaps with a Clp binding site, and the binding of Clp to the *engXCA* promoter could be outcompeted and displaced by HpaR1. Taken together, this is the first example of the activation of a single promoter by two global regulators in *Xcc*. Our transcriptomic data and bioinformatic analyses suggest that this regulatory interplay may occur at multiple promoters within the *Xcc* genome. There are very few reports of this type of regulation, but our findings suggest that this mechanism may occur more broadly than previously thought.

## Results

### Influence of HpaR1 on the global transcriptome of *Xcc*

In order to examine the regulatory influence of HpaR1, the impact of mutation of *hpaR1 *(ΔhpaR1) on the *Xcc* transcriptome was established by RNA sequencing (RNA‐seq). The transcriptome analysis, false discovery rate (FDR) ≤ 0.001 and log_2_ of the fold change (|log_2_FC|) ≥ 1 were considered for differentially expressed genes. The results showed that, of the 4273 annotated genes in *Xcc* strain 8004, 302 genes were found to be differentially expressed, 192 and 110 of which were up‐ and down‐regulated, respectively (Table S1, see Supporting Information). To verify the transcriptomic data, semi‐quantitative real‐time polymerase chain reaction (RT‐PCR) was performed to analyse the relative expression levels of several selected genes. The expression of these selected genes was consistent with the data from the transcriptome analyses (Table S2, see Supporting Information).

These regulated genes collectively were involved in a range of biological functions, including virulence, membrane transport, multidrug resistance, amino acid biosynthesis and signal transduction. Intriguingly, changes in the expression of genes involved in the activity of endoglucanase are consistent with previous findings (Su *et al.*, 2016). As the influence of the *clp* mutant on the *Xcc* transcriptome has been characterized previously (He *et al.*, 2007), we compared the general overlap of genes controlled by both HpaR1 and Clp. Forty‐two genes were found to be regulated by both HpaR1 and Clp (Tables 1 and S3, see Supporting Information). It was clear that there was extensive overlap in the genes involved in virulence. Importantly, changes in cellulose‐degrading endoglucanase encoded by *engXCA *(*XC_0639*) were observed in both mutants.

**Table 1:** HpaR1 is a global regulatory protein that affects the expression of a number of genes overlapping with the Clp protein.

Functional category	ORF number in strain 8004 (AT33913)	Gene name	Predicted product	Fold change (*hpaR1*‐/wt)	Putative HpaR1/Clp co‐binding sites
Cell envelope and cell structure	*XC_1459* (*XCC2658*)	*phuR*	Outer membrane haemin receptor	2.11	
Cellular processes	*XC_1410* (*XCC2704*)	*cheR*	Response regulator for chemotaxis	3.26	
	*XC_2234* (*XCC1952*)	*flgB*	Flagellar basal body rod protein FlgB	3.08	
	*XC_2237* (*XCC1949*)	*flgK*	Flagellar hook‐associated protein FlgK	2.18	
	*XC_2238* (*XCC1948*)	*flgL*	Flagellar hook‐associated protein FlgL	2.19	
	*XC_2243* (*XCC1943*)	*fliI*	Flagellar protein	2.38	
	*XC_2245* (*XCC1941*)	*fliK*	Flagellar protein	2.39	
	*XC_2263* (*XCC1923*)	*fliM*	Flagellar motor switch protein FliM	2.73	
	*XC_2267* (*XCC1919*)	*fliO*	Flagellar protein	2.18	
	*XC_2270* (*XCC1916*)	*fliP*	Flagellar biosynthesis protein FliP	2.24	
	*XC_2277* (*XCC1910*)	*flhB*	Flagellar biosynthesis protein FlhB	2.13	
	*XC_2278* (*XCC1909*)	*flhA*	Flagellar biosynthesis protein FlhA	2.33	
	*XC_2280* (*XCC1907*)	*fleN*	Flagellar biosynthesis switch protein	2.93	
Energy and carbon metabolism	*XC_0279* (*XCC0269*)		2,5‐Diketo‐d‐gluconate reductase B	–2.63	
	*XC_0281* (*XCC0271*)	*mocA*	Oxidoreductase	–2.80	GTGTGCGGAACGCTGAATCCACACC
	*XC_3683* (*XCC0549*)	*atpE*	F0F1 ATP synthase subunit C	–2.56	
Transport	*XC_1341* (*XCC2772*)	*fhuA*	TonB‐dependent receptor	2.16	
	*XC_1113* (*XCC3045*)	*bfeA*	Ferric enterobactin receptor	–2.15	
	*XC_2844* (*XCC1393*)	*brf*	Bacterioferritin	–2.07	
	*XC_3201* (*XCC1045*)		Bacterioferritin	–4.16	
	*XC_3293* (*XCC0942*)	*cysW*	Sulfate ABC transporter sulfate permease	–2.34	
Translation	*XC_0094* (*XCC0093*)	*tldD*	TldD protein	2.32	
	*XC_0096* (*XCC0094*)	*tldD*	TldD protein	2.53	GTGTTACTCGGTTTGCCCTGCGACAC
	*XC_0654* (*XCC3506*)		Prolyl oligopeptidase	2.37	GTGTCGCAGCGGCGGCGGGAACAC
	*XC_0667* (*XCC3494*)		ATP‐dependent protease peptidase	–2.34	
	*XC_1291* (*XCC2821*)	*hslV*	Endoproteinase Arg‐C	–2.72	
	*XC_1292* (*XCC2820*)		Endoproteinase Arg‐C	–3.47	
Pathogenicity and adaptation	*XC_1811* (*XCC2304*)	*acvB*	Virulence protein	2.04	
	*XC_3861* (*XCC3789*)	*acrA*	Acriflavin resistance protein	3.21	
	*XC_0639* (*XCC3521*)	*engXCA*	Major extracellular endoglucanase	–3.04	GTTTTCTGTGGGGACGATCACACCA
	XC_0026 (XCC0026)		Cellulase	–2.01	GTTTGTGAGCCCCTGCGCACATCA
	*XC_0027* (*XCC0027*)	*egl*	Cellulase	–4.53	
	*XC_1515* (*XCC2601*)	*egl*	Extracellular protease	–2.53	GTGTCAGCCGGCGACACACAGGCACCACA
	*XC_1658* (*XCC2454*)		GumB protein	–2.00	GTTCTATGCCATAGTGCACTGCAACACGCGA
	*XC_1664* (*XCC2448*)	*gumB*	GumH protein	–2.06	
	*XC_1667* (*XCC2445*)	*gumH*	GumK protein	–2.59	
	*XC_1668* (*XCC2444*)	*gumK*	GumL protein	–2.98	
	*XC_1669* (*XCC2443*)	*gumL*	GumM protein	–3.01	
	*XC_3590* (*XCC0645*)	*gumM*	Pectate lyase	–2.67	
	*XC_3591* (*XCC0644*)	*pel pel*	Pectate lyase	–16.11	GTGTCGCGGAAACTGAAAAGTACAC
Hypothetical proteins	*XC_0657* (*XCC3504*)		Hypothetical protein	2.79	
	*XC_1710* (*XCC2402*)		Hypothetical protein	3.22	
	*XC_2740* (*XCC1469*)		Hypothetical protein	13.84	

Note

False discovery rate (FDR) = 0.05 and absolute value of log_2_ of the fold change (log_2_FC) = 1 (equivalent to a fold change of two) were used as the cut‐off values. ‘+’ values represent genes up‐regulated in the *hpaR1* mutant, and ‘−’ values represent genes down‐regulated. The differentially expressed genes in the HpaR1 mutant were compared with those in the Clp mutant characterized by He *et al.* (2007). Forty‐two genes were found to be regulated by both HpaR1 and Clp.

The findings suggest that HpaR1 affects the expression of a larger number of genes than originally described, rather than having other roles in the bacterial cell. These findings also highlight that a subset of genes are controlled by both HpaR1 and Clp, in particular *engXCA *(*XC_0639*), although it is clear that both HpaR1 and Clp have independent actions on gene expression.

### HpaR1 positively regulates the expression of the endoglucanase major gene *engXCA*

**Figure 1:**
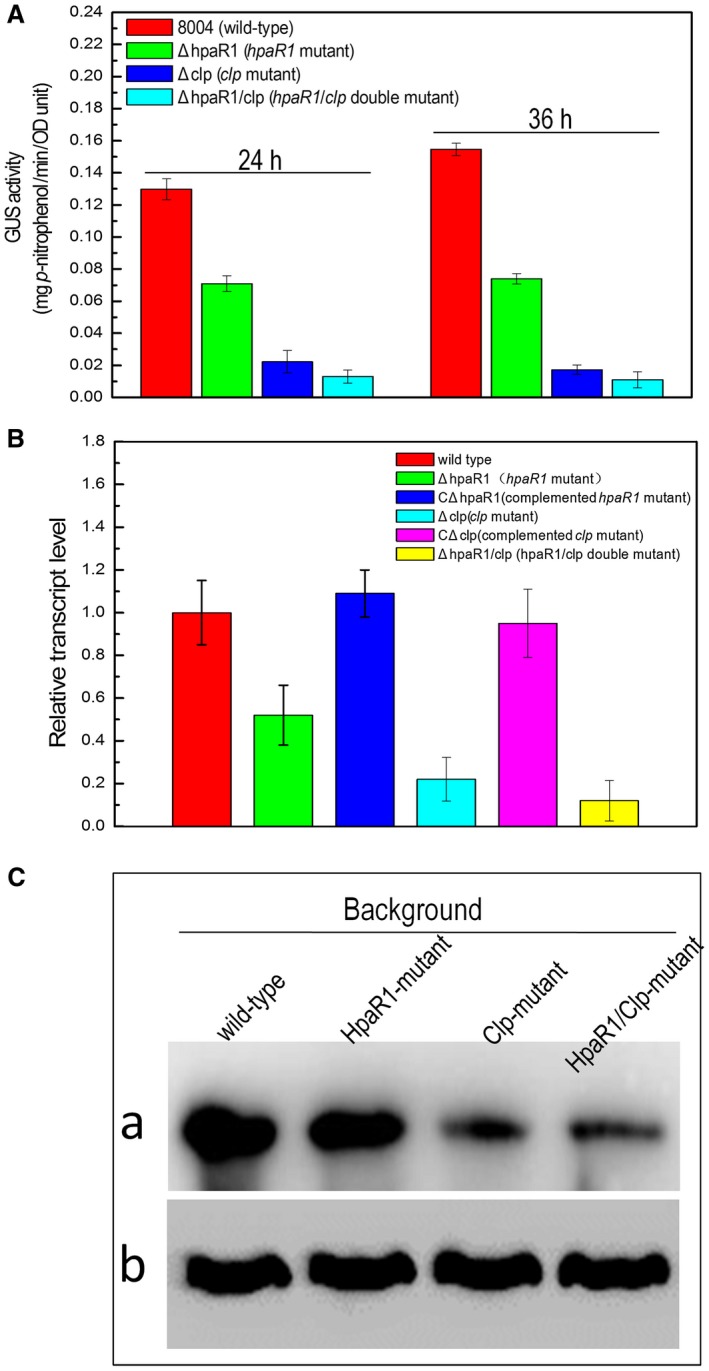
HpaR1 and Clp positively regulate the expression of *engXCA* in *Xanthomonas campestris* pv. *campestris* (*Xcc*)*. *(A) The expression of *engXCA* is affected positively by HpaR1 and Clp in NYG medium, as revealed by promoter reporter analysis. The graph shows the mean ± standard deviation (*n* = 3) of *engXCA* promoter‐GUS activity in the wild‐type strain 8004 compared with the HpaR1 mutant strain ΔhpaR1 and the Clp mutant strain Δclp*. *The experiment was repeated twice, and similar results were obtained. GUS, β‐glucuronidase. (B) The transcription level of *engXCA* in the *hpaR1 *or *clp* mutant could be restored to the wild‐type level by *in trans* expression of *hpaR1* or *clp*, as revealed by quantitative real‐time polymerase chain reaction (qRT‐PCR) analysis. RNA was isolated from cultures of *Xcc* strains grown in NYG medium for 24 h. The relative mRNA level was calculated with respect to the level of the corresponding transcript in the wild‐type strain 8004 (equal to unity). Values given are the means ± standard deviations of triplicate measurements from a representative experiment; similar results were obtained in two other independent experiments. (C) Mutation in HpaR1 or Clp reduces the *engXCA* product, compared with that in the wild‐type strain, as shown by western blot assay. *Xcc* strains chromosomally encoding EngXCA fused with the 6×His peptide tag in the wild‐type, HpaR1 deletion and Clp deletion backgrounds were created. The resulting strains 8004/EngXCA::6×His, ΔhpaR1/EngXCA::6×His and Δclp/EngXCA::6×His were cultured to an optical density at 600 nm (OD_600_) of 1.0, and total cell proteins were prepared; 30 μg of protein samples were separated by sodium dodecylsulfate‐polyacrylamide gel electrophoresis (SDS‐PAGE) and transferred to a polyvinylidene difluoride (PVDF) membrane. The presence of EngXCA protein was detected by an anti‐6×His monoclonal antibody (a). As a loading reference, the blot was also probed with an anti‐RNA polymerase β‐antibody (b). [Color figure can be viewed at wileyonlinelibrary.com]

HpaR1 and Clp have been shown previously to be required for the activity of endoglucanase in *Xcc* (He *et al.*, 2007; Su *et al.*, 2016). However, to date, only Clp has been shown to bind directly to the promoter of *engXCA*, controlling its expression. To explore the role of HpaR1 in the direct modulation of *engXCA* expression, we began by examining in detail the impact of an *hpaR1* mutation in *Xcc*. To do this, an *engXCA‐gus *reporter plasmid for transcriptional analysis was constructed. A 303‐bp DNA fragment harbouring the *engXCA *promoter sequence was amplified by PCR and cloned into the plasmid pL6*gus*, which harbours the promoterless *gusA *gene in the *Bam*HI/*Sph*I sites of pLARF6. The resulting reporter plasmid pGUSengXCA was introduced by triparental conjugation into the *hpaR1* deletion mutant strain ΔhpaR1 and the wild‐type strain 8004. The β‐glucuronidase (GUS) activities produced by the obtained transconjugant strains ΔhpaR1/pGUSengXCA and 8004/pGUSengXCA cultured in nutrient‐yeast‐glycerol (NYG) medium were then assessed. As shown in Fig. 1A, the GUS activity produced by strain ΔhpaR1/pGUSengXCA cultured for 24 and 36 h was reduced considerably when compared with that produced by strain 8004/pGUSengXCA grown at the same time.

The expression level of *engXCA* in the* hpaR1*‐deficient mutant was investigated by quantitative RT‐PCR (qRT‐PCR) analysis. The results showed that the *engXCA* transcript level in the mutant was decreased by 48% compared with the wild‐type when grown for 24 h in NYG medium (Fig. 1B). The reduced *engXCA* expression could be restored to the wild‐type level by *in trans* expression of *hpaR1*.

The *engXCA* product in the ΔhpaR1 strain was further estimated by western blot assay. This was achieved by chromosomally epitope tagging (6×His) the EngXCA proteins using homologous recombination (see Experimental procedures). The recombinant plasmid pK18*mobengXCA*H6, which was constructed by fusing the 6×His‐tag encoding sequence to the 3′ end of the *engXCA *gene and cloning the fused fragment into the suicide plasmid pK18*mobsacB*, was introduced into *Xcc* wild‐type strain 8004 and *hpaR1* deletion mutant strain ΔhpaR1, respectively. The resulting strains, 8004/EngXCA::6×His and ΔhpaR1/EngXCA::6×His, were used to test the EngXCA protein levels by western analysis. As shown in Fig. 1C, the EngXCA protein level in the ΔhpaR1/EngXCA::6×His strain was lower than that seen in the 8004/EngXCA::6×His strain, indicating that HpaR1 is important for the EngXCA protein level. Taken together, these findings reveal that disruption of *hpaR1* results in a significant reduction in the expression level of *engXCA*, indicating that HpaR1 positively regulates the expression of *engXCA*.

### HpaR1 binds directly to 25 nucleotides upstream of the *engXCA* promoter region

**Figure 2:**
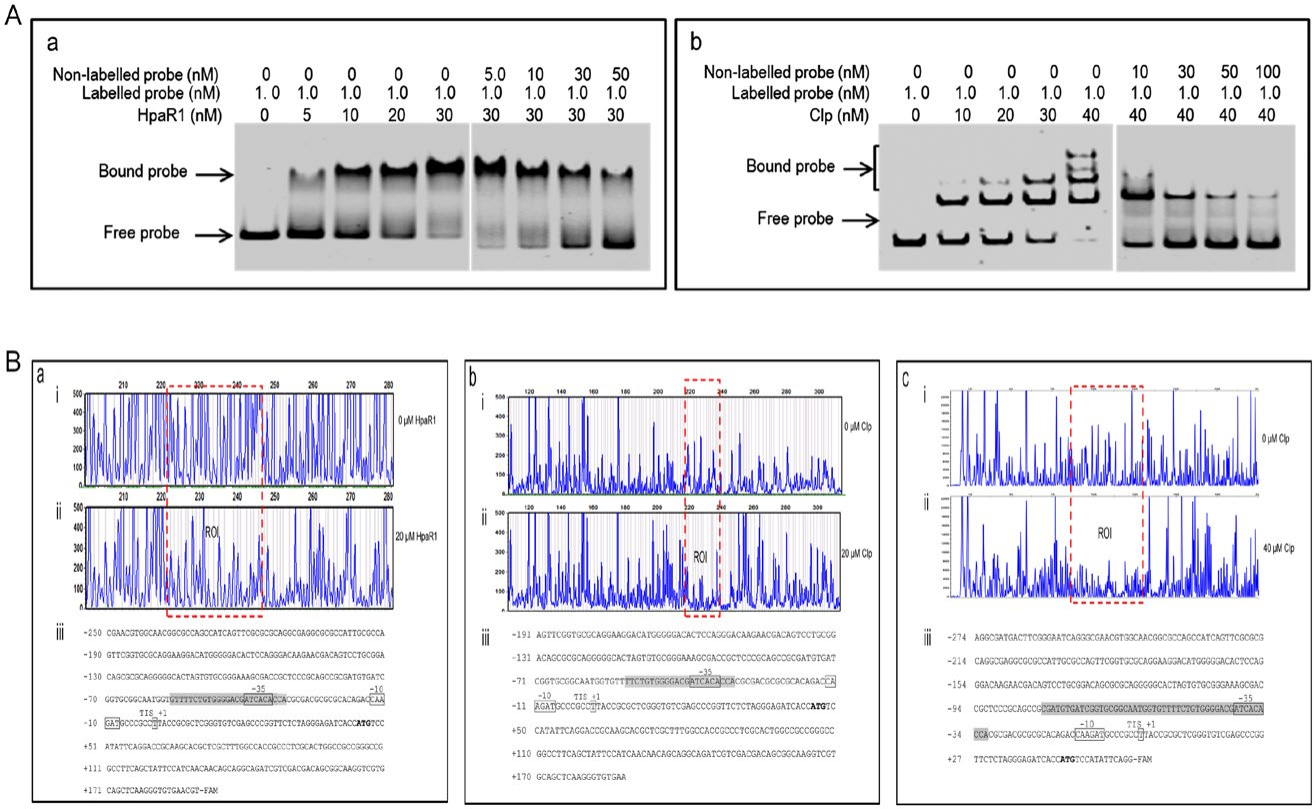
HpaR1 and Clp bind directly to the *engXCA* promoter. (A) Electrophoretic mobility shift assay (EMSA) of HpaR1 and Clp binding to the *engXCA* promoter fragments. The gel shift assay was carried out using the 6‐carboxyfluorescein (FAM)‐labelled DNA probe of the *engXCA* promoter, which spans from –256 to +47 relative to its transcription initiation site (TIS), and HpaR1 (a) or Clp (b) protein in a series of concentrations. DNA and protein were incubated at 30 ºC for 20 min before electrophoresis. The shifted band could be competed by excess of the unlabelled probe. The concentrations of HpaR or Clp, labelled probe and unlabelled probe are indicated. The migrated DNA–protein complexes and free probe are indicated by arrows. (B) Pinpointing of the binding sites of HpaR1 and Clp to the *engXCA* promoter. The HpaR1‐protected (a) and Clp‐protected (b, c) sequences were determined by dye primer‐based DNase I footprinting assay. Electropherograms show the protection pattern of the *engXCA* promoter after digestion with DNase I following incubation in the absence (i) or presence (ii) of HpaR1 or Clp; 20 μm of HpaR1, and 20 and 40 μm of Clp were used in DNase I footprinting assays. ROI, region of interest. (iii) *engXCA* promoter sequence with a summary of the DNase I footprinting assay results; 25‐bp HpaR1‐protected, and 22‐ and 49‐bp Clp‐protected sequences are highlighted with a grey background. Solid line squares indicate the TIS determined in strain 8004. Solid line rectangles indicate –35 and –10 elements predicted in this work.

To investigate whether transcriptional regulation of *engXCA* is achieved by the direct binding of HpaR1 to the promoter region, a 6×His‐tagged HpaR1 protein was first obtained as described previously (An *et al.*, 2011). The ability of purified 6×His‐tagged HpaR1 protein to bind to a 303‐bp DNA fragment encompassing the *engXCA *promoter (spanning nucleotides −256 to +47 relative to the transcription initiation site (TIS), named P_−256/+47_) was determined by EMSA (see Experimental procedures). The results revealed that the HpaR1 protein bound and therefore arrested the movement of the promoter sequence in the polyacrylamide gel (Fig. 2A‐a). The shifted bands could be competed by excess of the unlabelled probes, indicating that HpaR1 specifically binds to the *engXCA* promoter.

To identify the *engXCA* promoter region required for HpaR1 binding, five DNA fragments corresponding to nucleotide positions −184 to +47, −141/+47, −91/+47, −56/+47 and −36/+100 relative to the TIS of the *engXCA* promoter, and named P_−184/+47_, P_−141/+47_, P_−91/+47_, P_−56/+47_ and P_−36/+100_, respectively, were prepared by PCR amplification using 6‐carboxyfluorescein (FAM)‐labelled primers (see Experimental procedures). The binding of HpaR1 to these fragments was tested by EMSA (Fig. S1, see Supporting Information). The fragments spanning −184/+47, −141/+47, −91/+47 and −56/+47 nucleotides were found to be bound by HpaR1, whereas the fragment spanning −36/+100 nucleotides was not (Fig. S1B). The findings indicate that HpaR1 binding does not reside in the region spanning position −36/+100 relative to the TIS of the *engXCA* promoter, but is most probably present in the region −56/+47.

**Figure 3:**
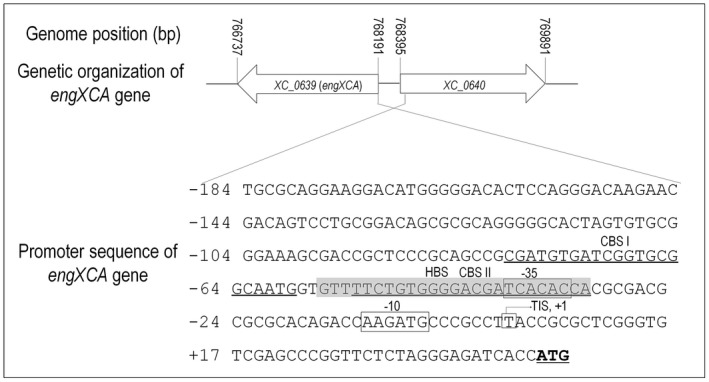
*engXCA *promoter sequence [–184 to +47 from the transcription initiation site (TIS)] with a summary of the DNase I footprinting assay results. The start codon of the gene *engXCA* is underlined. +1, transcription initiation site (TIS) determined by 5′‐rapid amplification of cDNA ends (5′‐RACE); –35 and –10, sequences resembling the sigma 70 promoter. HBS, HpaR1 binding site experimentally determined in this study, highlighted with a grey background. CBS I and CBS II, Clp binding sites experimentally determined in this study, indicated with underlines.

To determine the precise HpaR1 binding sequence in the *engXCA* promoter, a dye primer‐based DNase I footprinting assay was performed. A 440‐bp DNA fragment spanning nucleotides −250 to +190 relative to the TIS was amplified by PCR with the FAM‐labelled primer set Dye‐1F/R (see Experimental procedures) and incubated with 10 μm of 6×His‐tagged HpaR1 proteins. After 5 min of digestion with DNase I, the reaction was terminated and the digestion pattern was examined on a Applied Biosystems^™^ 3730 DNA Analyzer (Applied Biosystems, Waltham, MA, USA). By comparing the electropherograms with and without HpaR1 protein using GeneMarker software (SoftGenetics, PA, USA), a specific HpaR1‐protected region within the *engXCA *promoter was determined (Fig. 2B‐a). As shown in Fig. 2B‐a‐iii, the protected region consists of 25 nucleotides, which span from nucleotide −56 to −32 relative to the TIS (Fig. 3).

**Figure 4:**
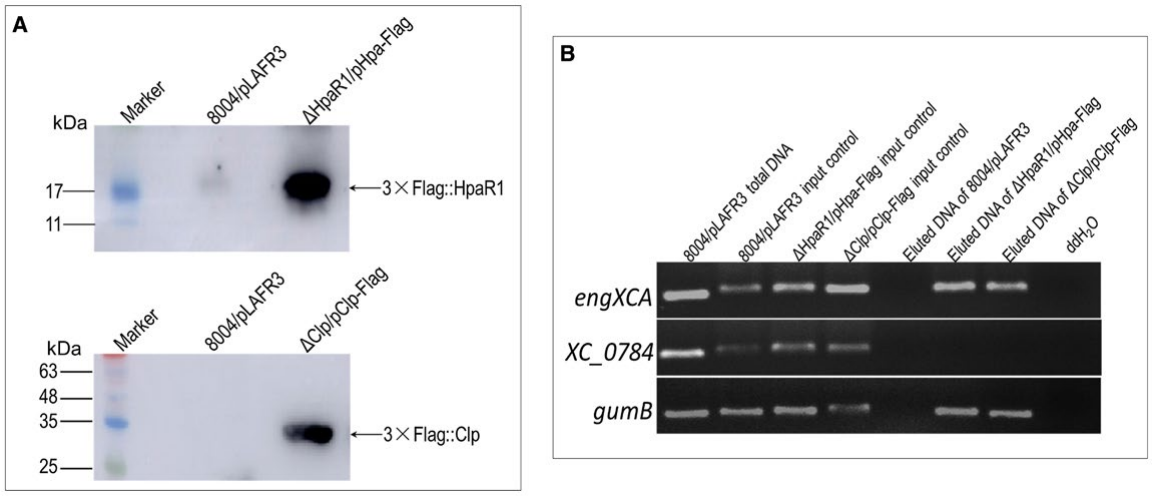
Chromatin immunoprecipitation (ChIP) assay showing that HpaR1 and Clp bind to the *engXCA* promoter region *in vivo*. *Xanthomonas campestris* pv. *campestris* (*Xcc*) strains encoding HpaR1 fused with the 3×Flag peptide and Clp fused with the 3×Flag peptide in the wild‐type background were created. The resulting strains ΔHpaR1/pHpa‐Flag and ΔClp/pClp‐Flag were cultured in NYG medium for 24 h and ChIP samples were prepared. Anti‐Flag was added to the ChIP samples, and incubated overnight. The bound DNA fragments and proteins were eluted. 8004/pLAFR3 was used as control strain. (A) Western blotting of the eluted 3×Flag::HpaR1 and 3×Flag::Clp fusion proteins. Protein samples were separated by sodium dodecylsulfate‐polyacrylamide gel electrophoresis (SDS‐PAGE) and transferred to a polyvinylidene difluoride (PVDF) membrane. The presence of the fusion proteins was detected by anti‐Flag monoclonal antibody. (B) Polymerase chain reaction (PCR) detection of eluted DNA. DNA fragments containing the *engXCA* promoter were PCR amplified using the eluted DNA from 3×Flag::HpaR1 or 3×Flag::Clp protein as template. Template DNA from a non‐conjugated ChIP sample was used as input control. Simultaneously, DNA fragments containing *gumB* and a cellulase S encoded gene *XC_0784* were amplified as positive and negative controls, respectively.

HpaR1 binding to the *engXCA* promoter *in vivo* was further tested by chromatin immunoprecipitation (ChIP) assay. A wild‐type background strain expressing the HpaR1 protein fused with 3×Flag‐tag (3×Flag::HpaR1) at the N‐terminus of HpaR1 was generated (Table S4, see Supporting Information). For this, a recombinant plasmid harbouring a DNA segment encoding 3×Flag tag fused to the 5′ end of the *hpaR1 *gene was introduced into the *hpaR1* deletion mutant strain. *Xcc* strains were grown in NYG medium for 24 h and used for the ChIP assay. A western blot assay showed that the 3×Flag::HpaR1 fusion protein could be eluted from the 3×Flag::HpaR1 expression strain ΔHpaR1/pHpa‐Flag, but not the control strain 8004/pLAFR3 (Fig. 4A). As illustrated in Fig. 4B, the result of ChIP assay showed that, using the eluted DNA from 3×Flag::HpaR1 protein as template, a PCR product was obtained by the primer pair designed for amplification of the DNA fragment containing the *engXCA *promoter, but no product could be obtained by the primers for the promoter of the *XC_0784* gene, indicating that the HpaR1 protein–*engXCA* promoter DNA complex exists in *Xcc* cells.

### Characteristics of the *engXCA* promoter relative to the HpaR1 binding site

Our work has revealed that HpaR1 binds upstream of the putative *engXCA *promoter ‘−35’ element (Fig. 2B‐a). To obtain a better understanding of the characteristics of the *engXCA *promoter in relation to HpaR1 binding, we carried out 5′‐rapid amplification of cDNA ends (5′‐RACE) analysis. Using this method, we determined the TIS of the *engXCA* promoter in *Xcc* 8004. As described previously (Su *et al.*, 2016), cDNA fragments corresponding to the 5′ end of the *engXCA* gene were amplified by PCR with nested gene‐specific primers and the anchor‐specific primer. These fragments were then cloned into the vector pMD19‐T and the resulting plasmids were sequenced. A high‐quality sequence was obtained from 21 recombinant clones, 17 of which showed the same transcriptional start nucleotide at position ‘−44’ (nucleotide T) upstream of the predicted translation ATG start codon (Fig. 3). These results revealed that the TIS of *engXCA* in strain 8004 is the ‘−44’ nucleotide T and is identical with that in *X. campestris* strain Xc17 (Hsiao *et al.*, 2005).

In parallel, we evaluated the predicted ְ‘−10’ (CAAGAT) and ‘−35’ (ATCACA) elements in the *engXCA *promoter region of strain 8004. To do this, two *engXCA* mutant promoters (spanning nucleotides −256 to +47 relative to TIS) with ‘CAAGAT’ and ‘ATCACA’ shifted to ‘CAAGCG’ and ‘ATCAGT’, respectively, were created by site‐directed mutagenesis (see Experimental procedures). The activity of reporter plasmid assays revealed that the promoter sequences with mutation in −10 or −35 elements produced significantly less GUS activity than the wild‐type promoter (Fig. S2A, see Supporting Information). *In vitro *transcription assays were further carried out with two *engXCA* mutant promoters (spanning nucleotides −191 to +126 relative to TIS) as template (see Experimental procedures). The results revealed that the mutant promoters generated low transcript levels compared with the wild‐type promoter (Fig. S2B). These findings suggest that ‘CAAGAT’ and ‘ATCACA’ are the −10 and −35 elements of the *engXCA* promoter, respectively.

### HpaR1 enhances the expression of *engXCA* via binding to its promoter

In general, transcription factors repress promoter activity by binding to operator sequences that overlap with the –10 or –35 elements of the promoter, thus blocking access of RNAP. Conversely, promoter activity is enhanced by the binding of the transcription factor upstream of the ‘−35’ element, which, in turn, supports the binding of RNAP.

**Figure 5:**
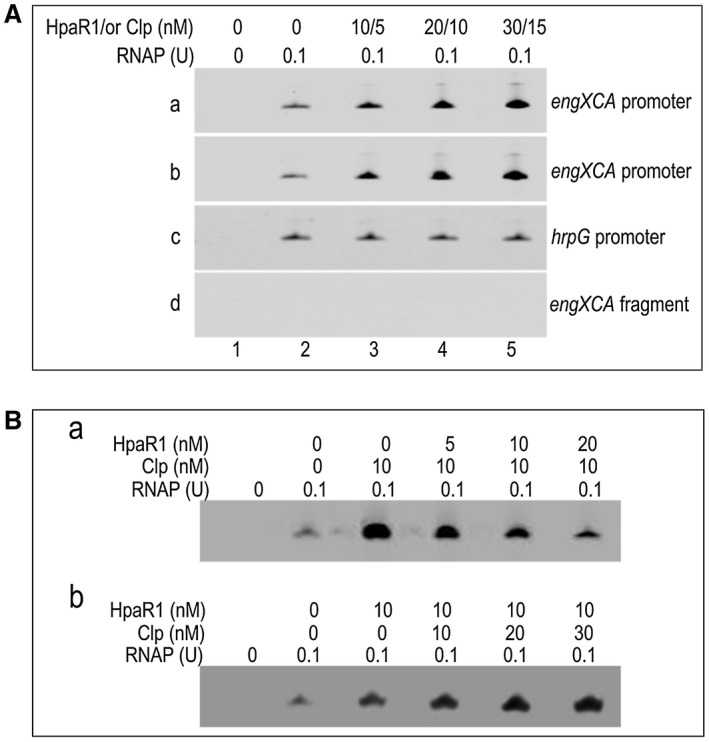
The role of HpaR1 and Clp on the transcription of the *engXCA* promoter *in vitro*. RNA was generated from a 317‐bp template DNA fragment extending from –191 to +126 relative to the transcriptional initiation site (TIS) of the *engXCA* promoter using *Escherichia coli *RNA polymerase (RNAP) holoenzyme. Transcription products were run on a 5% denatured polyacrylamide gel containing 7 m urea in 1 × Tris‐borate‐EDTA (TBE) electrophoresis buffer. (A) HpaR1 or Clp enhances the transcription of the *engXCA* promoter. Template DNA was incubated with various amounts of HpaR1 protein (a) or Clp (b) before the start of transcription by the addition of 0.1 U of RNAP. Transcription products (2 μL for HpaR1 and 1 μL for Clp) were then run. Lane 1, template DNA alone. Lane 2, template DNA with RNAP. Lanes 3–5, template DNA with RNAP and 10, 20 and 30 nm HpaR1 or 5, 10 and 15 nm Clp. A template DNA fragment containing the *hrpG* promoter (c) and a 126‐bp template DNA fragment extending from +1 to +126 relative to the TIS of the *engXCA* promoter (d) were used as controls. The amounts of RNAP and HpaR1 or Clp used are indicated at the top. (B) HpaR1 reduces the activation of Clp on the transcription of the *engXCA* promoter*.* Reactions were carried out with DNA fragments of the *engXCA* promoter and Clp and an enhancing amounts of HpaR1 (a), and HpaR1 and an enhancing amounts of Clp proteins (b). The amounts of proteins used are indicated above the photographs.

To understand how HpaR1 potentially enhances the expression of the *engXCA* gene, an *in vitro* transcription assay was first carried out. For this, 317‐bp template DNA fragments, corresponding to nucleotide positions −191 to +126 relative to TIS of the *engXCA* promoter, were incubated with RNAP holoenzyme from *Escherichia coli* with increasing amounts of purified 6×His‐tagged HpaR1 protein. The results showed that, although *engXCA* transcripts could be generated without HpaR1 protein, the *engXCA* transcription level was significantly increased when HpaR1 protein was added to the reaction (Fig. 5A‐a). Importantly, the use of the DNA fragment from the *hrpG* promoter showed no enhanced *hrpG* transcriptional level when used in the same assay (Fig. 5A‐c). These data demonstrate that HpaR1 specifically enhances the transcription of the *engXCA *promoter, which is consistent with our reporter and qRT‐PCR findings.

**Figure 6:**
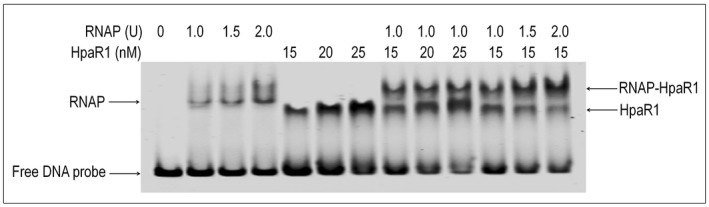
Electrophoretic mobility shift assay reveals that HpaR1 protein enhances the binding of RNA polymerase (RNAP) to the *engXCA* promoter. The gel shift assay was carried out using the two proteins, RNAP and HpaR1, with a 6‐carboxyfluorescein (FAM)‐labelled DNA probe of the *engXCA* promoter which spans from −191 to +126 relative to its transcriptional initiation site (TIS) (2 nm). RNAP and/or HpaR1 was added as indicated above.

These findings were corroborated by a set of EMSAs that estimated the effect of HpaR1 on the binding of RNAP to the *engXCA *promoter. For these experiments, a 317‐bp FAM‐labelled DNA fragment containing the *engXCA* promoter was generated. As shown in Fig. 6, a single band shift was identified, whose intensity was enhanced with the addition of increasing concentrations of either RNAP or HpaR1. Interestingly, the presence of both RNAP and HpaR1 in the assay led to an even greater band shift (Fig. 6). The amount of the RNAP–HpaR1–*engXCA *promoter complex was greater than that of the RNAP–*engXCA *promoter complex, although they contained the same amount of RNAP. Moreover, the amount of the RNAP–HpaR1–*engXCA *promoter ‘complex’ was increased in intensity with increasing concentrations of RNAP. These observations suggest that both RNAP and HpaR1 proteins bind together to the *engXCA *promoter DNA, and that RNAP molecules are recruited to the *engXCA *promoter when HpaR1 is present.

To further explore the impact of HpaR1 binding on the expression of the *engXCA *gene, 13 nucleotides (GTTTTCTGTGGGG) within the HpaR1 binding sequence determined by footprinting assay were deleted by site‐directed mutagenesis. The labelled 175‐bp (from −141 to +47 relative to TIS) *engXCA *promoter fragments with a 13‐nucleotide deletion were incubated with HpaR1 and then analysed by EMSA (Fig. S3A‐i, see Supporting Information). The results show that the deletion diminished the binding of HpaR1, indicating that these nucleotides contribute to HpaR1 binding.

The effect of the mutation on the promoter activity of *engXCA* was further estimated using *in vitro* transcription assay. To do this, a 304‐bp DNA fragment (spanning nucleotides −191 to +126 relative to TIS of the *engXCA* promoter) with a 13‐nucleotide deletion within the HpaR1 binding site was constructed (see Experimental procedures). *In vitro* transcription assays were performed with the mutated and wild‐type promoter as templates. Compared with the wild‐type promoter, the use of the mutated promoter showed no obvious increase in the *engXCA* transcription level when HpaR1 protein was added to the reaction (Fig. S3B‐i). These combined data demonstrate that HpaR1 binds to the promoter of *engXCA*, and that an absence of part of the binding sequence can impede HpaR1 binding and the activation of *engXCA *transcription.

### Clp binds to two sites on the *engXCA* promoter with one overlapping with the site targeted by HpaR1

As discussed above, the Clp protein is a homologue of the global regulator CRP [cyclic adenosine monophosphate (cAMP) receptor protein] of *E.* *coli*. However, in *Xcc*, mutation in *clp* causes a reduction in the production of exopolysaccharide and the activity of extracellular enzymes, and a loss of virulence (Chin *et al.*, 2010; de Crecy‐Lagard *et al.*, 1990; Tao *et al.*, 2010). In the *Xcc* strain Xc17, it was predicted that there were two tandem CBSs, named CBS I and CBS II, within the *engXCA *promoter (Hsiao *et al.*, 2005). However, the same study only showed evidence of Clp binding to CBS II overlapping with the −35 element, but not CBS I, which is located upstream of the −35 element (Hsiao *et al.*, 2005). Subsequent studies in *Xcc* strain 8004 also only showed one functional binding site for Clp (Chin *et al.*, 2010; Tao *et al.*, 2010).

Our assessment of the Clp and HpaR1 binding sites on the *engXCA *promoter suggests that they overlap, which is not a commonly reported phenomenon in bacteria. To confirm that Clp and HpaR1 truly have overlapping binding sites on the *engXCA* promoter, a set of EMSAs was carried out.

First, to test Clp binding of the *engXCA* promoter, a 6×His‐tagged Clp protein was constructed and expressed in *E. coli*, and increasing amounts of purified Clp were incubated with the FAM‐labelled 303‐bp DNA fragment of the *engXCA* promoter (spanning nucleotides −256 to +47 relative to TIS). Here, it was observed that the addition of Clp at a concentration of 10 nm resulted in a slower mobility of the 303‐bp DNA fragment in the assay. However, there were further band shifts observed when high concentrations of Clp were used (Fig. 2A‐b). This differed from the results shown by Hsiao *et al.* (2005) for the Xc17 strain. This observation may be explained by the original theory, i.e. the *engXCA* promoter contains two Clp binding sites (Hsiao *et al.*, 2005). To investigate whether Clp binds to two sites in the *engXCA* promoter, a series of DNA fragments (P_−184/+47_, P_−141/+47_, P_−91/+47_, P_−56/+47_ and P_−36/+100_) was generated to test the binding of Clp. In these EMSAs, Clp protein at a concentration of 20 or 30 nm was used. As shown in Fig. S1C, two retarded bands were present in the assay with the fragments spanning −184/+47, −141/+47 or −91/+47 nucleotides relative to TIS. However, only one retarded band appeared with the fragment spanning −56/+47 nucleotides, and no retarded band with the fragment corresponding to −36/+100 nucleotides. Therefore, the data suggest that one Clp binding site exists in the region of nucleotide positions −91 to −56 relative to TIS, whereas the other is located in the region between −56 and −36 nucleotides relative to TIS.

To locate specifically the CBSs within the *engXCA* promoter, dye primer‐based DNase I footprinting assays were carried out with a 378‐bp FAM‐labelled DNA fragment of the *engXCA *promoter (spanning nucleotides −191 to +187 relative to TIS) and 20 μm Clp protein. As shown in Fig. 2B‐b, only one Clp‐protected region on the *engXCA *promoter was found. This protected region, consisting of 22 nucleotides spanning positions −53 to −32 relative to TIS (Fig. 2B‐b‐iii), is identical with CBS II predicted by Hsiao *et al.* (2005). DNase I footprinting assays were further carried out with a 333‐bp FAM‐labelled DNA fragment (spanning nucleotides −274 to +59 relative to TIS) and 40 μm Clp protein (Fig. 2B‐c). Interestingly, an expanded Clp‐protected region, spanning positions −80 to −32 relative to TIS, was found (Fig. 2B‐c‐iii), implying that Clp, in addition to CBS II, binds to CBS I consisting of nucleotides from −80 to −59 relative to TIS (Fig. 3).

To further verify the binding of Clp to the two sites in the *engXCA *promoter, 10 nucleotides (TGTGATCGGT) within CBS I were deleted by site‐directed mutagenesis (see Experimental procedures). The obtained labelled 178‐bp *engXCA *promoter fragments (from −141 to +47 relative to TIS) with a 10‐nucleotide deletion within CBS I, and the above labelled 175‐bp *engXCA* promoter fragments (with 13 nucleotides of the HpaR1 binding site or 10 nucleotides of the CBS II deletion) were incubated with Clp and analysed by EMSA. The results showed that mutations within the two Clp binding sequences clearly interfered with the Clp−Clp−*engXCA* promoter complex, implying that the mutations prevent Clp from binding to the corresponding site, whereas Clp still binds to the innate binding site (Fig. S3A‐ii, iii). Taken together, these findings demonstrate that Clp can bind to both CBS I and CBS II on the *engXCA *promoter, but prefer to bind to CBS II overlapping with the HpaR1 binding site.

The existence of the *in vivo* Clp protein–*engXCA* promoter DNA complex was further identified by ChIP assay with a strain expressing the Clp protein fused with a 3×Flag‐tag (3×Flag::Clp) at the N‐terminus of Clp (ΔClp/pClp‐Flag) (Table S4). As shown in Fig. 4B, the result of ChIP assay revealed that, using the eluted DNA from the 3×Flag::Clp protein as template, a DNA fragment of the *engXCA* promoter was obtained, indicating the binding of Clp to the *engXCA* promoter *in vivo*.

### HpaR1 displaces Clp on the *engXCA* promoter

The fact that HpaR1 and Clp overlap in the *engXCA* promoter prompted us to investigate the effect of both HpaR1 and Clp on the transcription of the *engXCA* promoter. To do this, we first evaluated the role of Clp on the expression of *engXCA* in *Xcc* strain 8004. As shown in Fig. 1, mutation in Clp reduced the expression level of *engXCA* greatly, indicating that Clp is a key regulator for the expression of *engXCA*. In addition, the expression level of *engXCA* in the double mutant of HpaR1/Clp seemed to be lower than that in the Clp deletion mutant.

Then, we ascertained the regulation of Clp on the expression of *engXCA* as a result of Clp binding. An *in vitro* transcription assay was performed with a 317‐bp DNA fragment (nucleotides spanning positions −191 to +126 relative to TIS on the *engXCA* promoter) as template and increasing amounts of Clp proteins. As shown in Fig. 5A‐b, the addition of Clp protein increased the *engXCA* transcription level, indicating that Clp enhances *engXCA* transcription *in vitro*. In addition, *in vitro* transcription assays with the mutated *engXCA* promoter (10 nucleotides deleted within the Clp binding sequences) were also carried out (see Experimental procedures). The results revealed that mutation in CBS II, rather than CBS I, reduced Clp activation (Fig. S3B‐ii, iii).

The effects of both HpaR1 and Clp on *engXCA* transcription were then investigated. An *in vitro* transcription assay was performed by the addition of 10 nm of Clp and varied amounts of HpaR1. As shown in Fig. 5B‐a, the Clp protein alone increased transcription; when HpaR1 was added, the transcription level of *engXCA* was reduced, indicating that HpaR1 represses Clp activation of *engXCA*. An experiment with the addition of 10 nm of HpaR1 with increasing amounts of Clp was further performed (Fig. 5B‐b). HpaR1 alone also increased the transcription level of *engXCA*. Interestingly, slight enhancement of the transcription level was observed in the presence of Clp, indicating that Clp might play impacts on HpaR1 activation (see Fig. 5B‐b). These data indicate that activation by HpaR1 and Clp is intertwined, although each activator is more potent in the absence of the other.

**Figure 7:**
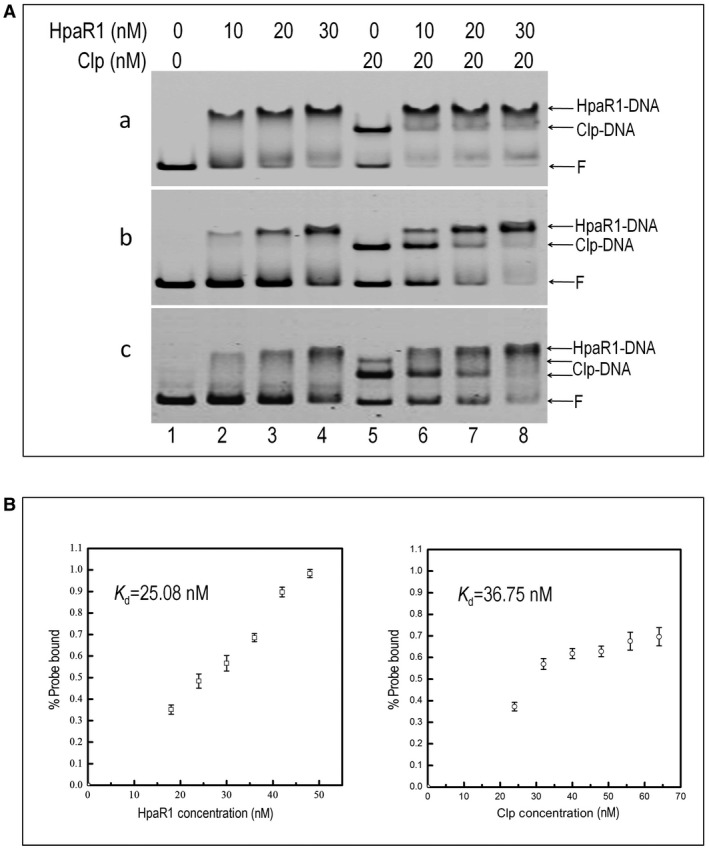
Electrophoretic mobility shift assays (EMSAs) testing the binding affinity of HpaR1 and Clp with the *engXCA* promoter. (A) HpaR1 dislodges Clp binding to the *engXCA* promoter. EMSAs were carried out with 1 nm of 6‐carboxyfluorescein (FAM)‐labelled DNA fragment incubated with Clp and HpaR1 simultaneously for 40 min (a), or Clp for 20 min first, and then with HpaR1 for another 20 min (b, c). Samples were run on 6% polyacrylamide‐Tris‐borate‐EDTA (TBE) gel. The amounts of HpaR1 and Clp used in each reaction are indicated at the top. F, free DNA probe; Clp‐DNA, Clp–DNA complex; HpaR1‐DNA, HpaR1–DNA complex. (a) 184‐bp DNA fragment (–64 to +120) containing CBS II and HBS incubated with Clp and HpaR1 simultaneously. (b) 184‐bp DNA fragment (–64 to +120) containing CBS II and HBS incubated with Clp and HpaR1 in sequence. (c) 188‐bp DNA fragment (–141 to +47) containing two Clp binding sites, CBS I and CBS II, and HBS incubated with Clp and HpaR1 in sequence. (B) Quantification of binding affinities. EMSAs with various concentrations of HpaR1 or Clp were performed in triplicate. ImageJ software was used to quantify the amount of DNA bound to HpaR1 or Clp. The dissociation constant (*K*_d_) was calculated. Standard deviations are marked by error bars.

To gain further insights into the activation of *engXCA* by HpaR1 and Clp, it was investigated further whether or not HpaR1 binding to the *engXCA *promoter interferes with the binding of Clp. To do this, a competition EMSA was first performed with the addition of different ratios of HpaR1/Clp at the same time. In this experiment, a labelled 184‐bp *engXCA* promoter DNA fragment (−64 to +120) containing the HpaR1 and Clp overlapping binding sites was used for incubation with HpaR1 and/or Clp for 40 min, followed by separation using electrophoresis. As shown in Fig. 7A‐a (lane 5), in the presence of Clp at a concentration of 20 nm, approximately two of three DNA molecules formed the Clp−DNA complex; however, when both HpaR1 and Clp were present simultaneously in the reaction, only the HpaR1–DNA complex appeared, but not the Clp–DNA or HpaR1−Clp−DNA complexes (Fig. 7A‐a), indicating that HpaR1 binds more efficiently to the *engXCA* promoter compared with Clp, and probably interferes with Clp binding.

We then tested the 184‐bp *engXCA* promoter DNA fragments incubated with 20 nm Clp for 20 min to form a complex consisting of a Clp molecule and a DNA molecule. Different concentrations of HpaR1 were added and incubated for 20 min (Fig. 7A‐b). At 20 nm Clp (lane 5), and on addition of 10 nm HpaR1 (lane 6), a clear HpaR1–DNA complex was observed. On addition of 20 nm HpaR1 (lane 7), most of the DNA probe was bound by HpaR1, whereas the DNA probe bound by Clp was strongly reduced compared with that on addition of 20 nm Clp only. A labelled 188‐bp *engXCA* promoter DNA fragment (−141 to +47) containing two Clp binding sites was further used for EMSA (Fig. 7A‐c). After incubation with 20 nm Clp, a band representing two Clp proteins bound to the *engXCA* promotor was seen (lane 5). The addition of 10 nm HpaR1 to this assay resulted in the appearance of an HpaR1–DNA complex and the disappearance of the Clp–Clp–DNA complex (lane 6). Interestingly, when a concentration of 30 nm HpaR1 was used, only the HpaR1–DNA complex was observed (lane 8). This suggests that Clp does not target CBS I when HpaR1 precludes Clp from binding to CBS II.

To further compare the binding affinities of HpaR1 and Clp to the *engXCA* promoter, the quantification of EMSA was performed with a 184‐bp DNA fragment (−64 to +120) containing CBS II and the HpaR1 binding site, and a range of HpaR1 and Clp concentrations. As shown in Fig. 7B, HpaR1 and Clp bound to the *engXCA* promoter with dissociation constants (*K*_d_) of 25.08 and 36.75 nm, respectively, indicating strong affinity binding of HpaR1 compared with Clp.

Overall, these data indicate that both HpaR1 and Clp proteins specifically bind to the *engXCA* promoter. Furthermore, under the conditions tested, it appears that HpaR1 has a higher affinity for binding the *engXCA *promoter as it outcompetes Clp binding to its target site. However, in this study, although Clp acting alone in the *engXCA* promoter generates a higher promoter activity than that when both Clp and HpaR1 proteins act together, HpaR1 was observed to dislodge Clp binding in the *engXCA* promoter.

## Discussion

During infection, bacteria control gene expression in a multitude of ways in response to alterations in their surroundings. One of the most deeply studied mechanisms is a bacterium’s use of transcriptional regulators. Although bacteria encode large numbers of transcription factors, there has been limited investigation into how combinations of transcriptional regulators control gene expression coordinately, given that many appear to co‐regulate genes involved in microbial pathogenesis. The data generated here describe how two global transcriptional regulators, HpaR1 and Clp, co‐regulate a subset of virulence genes in *Xcc*. The work reveals an interesting mechanism in which HpaR1 and Clp both bind specifically to the promoter of *engXCA* to positively control its expression. Interestingly, the binding sites for both proteins overlap, suggesting that they compete for binding and control of *engXCA* expression. Further analysis shows that, under the conditions tested, HpaR1 has a higher affinity for binding to the *engXCA* promoter.

There are very few cases in which multiple transcriptional regulators control gene expression by binding to a promoter at overlapping sites. These include the regulators Lrp and ArgP from *E. coli*, and AfsR and PhoP from *Streptomyces *species, which have been shown to act as competitive activators by binding to sites that overlap on their respective promoter targets (Peeters *et al.*, 2009; Santos‐Beneit *et al.*, 2011). Despite the lack of *in vivo *evidence in our work, we suppose that there is a difference in mechanism acting here, as Clp and HpaR1 co‐regulate the expression of the *engXCA* gene in alternative ways. The evidence supporting this proposal was found when Clp was shown *in vitro* to bind independently to the *engXCA* promoter to generate a higher activity than that observed when both Clp and HpaR1 proteins acted together. This is very much in contrast with the Lrp/ArgP and AfsR/PhoP systems which appear to function in a competitive manner.

Using the VB search algorithm, we gained some insights into how broadly this competitive HpaR1 and Clp regulation mechanism is used in *Xcc*. By examination of the other genes potentially controlled directly by both HpaR1 and Clp (Table 1), we identified several HpaR1 binding sites that overlapped with the putative Clp binding site [motif ‘GTGT(N15‐N20)ACAC’] (He *et al.*, 2007), suggesting that they might be controlled by the same mechanism. These included *XC_3591* encoding pectate lyase and *XC_1515* encoding extracellular protease (Table 1). This suggests that, although this type of regulation appears to be uncommon, this may be a result, in part, of the phenomenon being difficult to identify and characterize. Therefore, it is likely to occur more broadly than believed in both *Xcc* and other bacteria.

Previously, Hsiao *et al.* (2005) have described how Clp binds to the *engXCA* promoter to activate gene expression. However, despite their assessment, they did not determine or provide evidence of the precise site(s) of Clp binding. Although they proposed two sites of binding (CBS I and CBS II), they discounted the first, as they could not provide evidence of Clp binding to this region of the *engXCA* promoter. In the current study, we have shown that Clp binds to both sites of the *engXCA* promoter, which is dependent on the concentration of Clp. The CBS I site is a low‐affinity site that requires high concentrations of Clp to observe binding, whereas CBS II is a high‐affinity site where binding is seen at low concentrations of Clp protein. Furthermore, the presented data suggest that the CBS I site spans nucleotides −80 to −59 relative to TIS, and the CBS II site spans nucleotides −53 to −32 relative to TIS.

**Figure 8:**
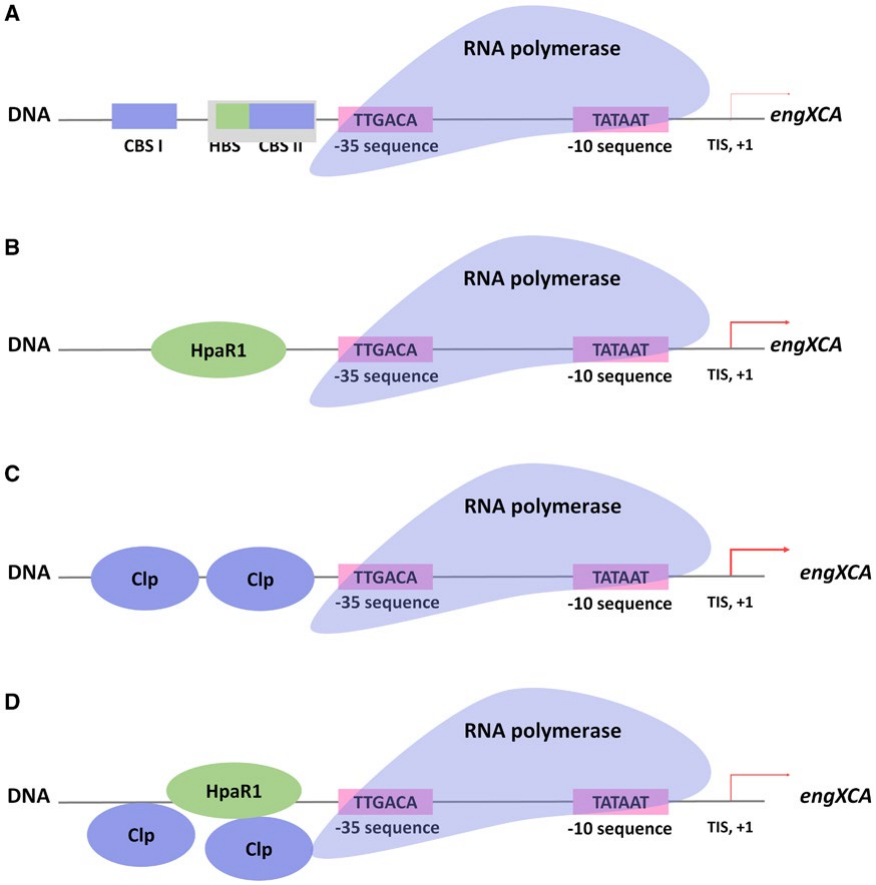
Model of regulation of *engXCA* in *Xanthomonas campestris* pv. *campestris* (*Xcc*) by HpaR1 and Clp. HpaR1 and Clp are global transcriptional regulators that influence the expression of many of the same genes, including *engXCA*, by binding directly to the promoter. (A) The *engXCA *promoter encodes a HpaR1 binding site (HBS) and two Clp binding sites (CBS I and CBS II). (B) HpaR1 binding to the single HBS on the *engXCA* promoter. In this scenario, *engXCA *expression is considered to be high. (C) Clp binding to the two Clp binding sites (CBS I and CBS II) on the *engXCA* promoter. In this scenario, *engXCA *expression is considered to be high. (D) Both HpaR1 and Clp compete for binding to the *engXCA* promoter. HpaR1 outcompetes Clp for HBS which overlaps with CBS II, but it is likely that Clp binds to CBS I. In this scenario, the resulting *engXCA* expression is considered to be lower than when each protein binds in the absence of the other. [Color figure can be viewed at wileyonlinelibrary.com]

Examination of the gene expression and protein binding data generated during this investigation, together with data from previous studies, allowed us to generate a model for how HpaR1 and Clp participate in the alteration of *engXCA* gene expression observed when *Xcc* is grown in laboratory medium (Fig. 8). Our findings demonstrate that a significant overlap exists between the HpaR1 and Clp transcriptional regulatory systems, and adds to the understanding of the molecular mechanisms used by *Xcc* to alter gene expression and the proteins encoded in response to environmental changes. Interestingly, *Xcc* HpaR1 and Clp binding sites can be proximal, suggesting extensive interplay. However, further work is required to address many questions left outstanding, such as how broadly distributed is this regulatory phenomenon, are other genes, such as *XC_*3591 and XC_*1515*, which contain the HpaR1 binding site and CBSs regulated in the same fashion, and does the affinity of Clp and HpaR1 binding to the *engXCA *promotor change with changes in the environment? Furthermore, it has been demonstrated recently that targeting bacterial virulence factor regulation during infection can decrease infection severity. The data presented suggest that dual targeting of HpaR1 and Clp simultaneously may be a better potential approach for the development of novel antimicrobials.

## Experimental Procedures

### Bacterial strains, plasmids and growth conditions

The bacterial strains and plasmids used in this study are listed in Table S4. The *E. coli *strains were grown in Luria–Bertani medium (Miller, 1972) at 37 °C, and the *Xcc *strains were grown at 28 °C in NYG (nutrient‐yeast‐glycerol) medium (Daniels *et al.*, 1984). Antibiotics were added at the following concentrations as required: kanamycin (Kan), 25 μg/mL; rifampicin (Rif), 50 μg/mL; ampicillin (Amp), 100 μg/mL; spectinomycin (Spc), 50 μg/mL; tetracycline (Tet), 5 μg/mL for *Xcc *and 15 μg/mL for *E. coli*.

### DNA and RNA manipulations

The DNA manipulations followed the procedures described by Sambrook *et al.* (1989). Conjugation between the *Xcc *and *E. coli* strains was performed as described by Turner *et al.* (1985). The restriction endonucleases, T4 DNA ligase and *pfu* polymerase were provided by Promega (Shanghai, China). The total RNAs were extracted from cultures of the *Xcc *strains using a Total‐RNA Extraction Kit (Promega) according to the manufacturer’s instructions. RNA was reverse transcribed by Superscript II reverse transcriptase (Invitrogen, Waltham, MA, USA), according to the manufacturer’s protocol. For semi‐quantitative RT‐PCR, the resulting cDNA was diluted and used with specific primers (Table S5, see Supporting Information). Relative quantification of gene expression was performed using the 16S rRNA gene as a control. qRT‐PCR testing of the transcription level of *engXCA* was conducted with total RNA extracted from *Xcc* strains grown in NYG medium for 24 h. The Synergy brand (SYBR) green‐labelled PCR fragments were amplified using the primer set engXCA‐F/R (Table S5), which was designed from the transcribed region of *engXCA*. The relative mRNA level was calculated with respect to the level of the corresponding transcript in the wild‐type strain 8004 (equal to unity). The expression level of the 16S rRNA gene was used as an internal standard. The qRT‐PCR tests were performed in triplicate.

### Construction of promoter reporter plasmid

A promoter reporter plasmid for *engXCA* was constructed by fusing a 303‐bp DNA fragment upstream of the *engXCA* open reading frame (ORF) (including the translation start codon ATG) with the promoterless GUS‐encoding ORF (excluding the translation start codon ATG). The *engXCA* promoter of the *Xcc* wild‐type strain 8004 was amplified with the primer set P_engXCA_‐1F/R (Table S5). Primers were modified to give *Eco*RI‐ or *Bam*HI‐compatible ends (underlined) (Table S5). The DNA fragment of the *gusA *coding region was obtained as described previously (Su *et al.*, 2016). The two fragments obtained were cloned into the promoterless cloning sites of the plasmid pLAFR6 to generate the reporter plasmid named pGUS*engXCA* (Table S4).

### Construction of reporter strain

To construct a chromosomally encoding EngXCA::6×His, PCR was employed to create a sequence encoding an in‐frame 6×His peptide at the C‐terminus of EngXCA with the primer set O_engXCA_‐F/R. This sequence was cloned into the suicide vector pK18mob*sacB* (Schäfer *et al.*, 1994), and the resulting plasmid, named pK18*mobengXCA*H6, was introduced into *Xcc* wild‐type strain 8004, *hpaR1* deletion mutant strain ΔhpaR1 and *clp* deletion mutant strain Δclp by triparental conjugation. The transconjugants were screened on selective agar plates containing 5% sucrose. The insertion of the 6×His tag at the C‐terminal end of the chromosomally encoded *engXCA* gene was verified by both PCR and DNA sequencing, and the obtained strains encoding EngXCA::6×His in the wild‐type, HpaR1 mutant, Clp mutant and HpaR1/Clp mutant backgrounds were named 8004/EngXCA::6×His, ΔhpaR1/EngXCA::6×His, Δclp/EngXCA::6×His and HpaR1/Clp/EngXCA::6×His, respectively.

### Determination of TIS

To determine TIS of the *engXCB* gene, the 5′‐RACE method was carried out with the *engXCB* sequence‐specific primer *eng*RT1‐3 (Table S5). The assay was performed as described previously (Li *et al.*, 2014). Briefly, total cellular RNA was extracted from the *Xcc* strains grown in NYG medium to an optical density at 600 nm (OD_600_) of 1.0. cDNA fragments were obtained using the 5′‐RACE Kit (Invitrogen), and the PCR products were cloned into the pMD19‐T vector and sequenced.

### Western blotting

Western blotting followed the procedure described by Sambrook *et al.* (1989). Bacterial proteins separated by sodium dodecylsulfate‐polyacrylamide gel electrophoresis (SDS‐PAGE) were electrotransferred onto a polyvinylidene difluoride (PVDF) membrane (Millipore, Billerica, MA, USA). After blocking with 1% milk, the proteins in the membrane were incubated with the 1 : 1500 diluted or anti‐His‐tag anti‐Flag‐tag mouse monoclonal antibody as the primary antibody, followed by washing four times with TBST buffer [Tris, 20 mm; NaCl, 0.3 m; Tween‐20, 0.08% (v/v)]. The diluted 1 : 2000 horseradish peroxidase (HRP)‐conjugated goat anti‐mouse immunoglobulin G (IgG) was used as the secondary antibody. After washing the membrane four times, the luminescence signal was detected according to the manufacturer’s instructions. For a loading control, proteins were probed with the anti‐RNAP β‐antibody (EPR18704; Abcam, Cambridge, UK) at 1 : 2000 dilution as primary antibody, and the HRP‐conjugated goat anti‐rabbit IgG H&L (31 460; Thermo Scientific Waltham, MA, USA) at 1 : 5000 dilution as secondary antibody.

### Overproduction and purification of proteins

To overproduce the 6×His‐tagged form of Clp, the 690‐bp ORF was amplified by PCR from the genomic DNA of strain 8004 using the primers Clp‐OF/R. The primers were modified to give *Bam*HI‐ or *Hin*dIII‐compatible ends. After confirmation by sequencing, the amplified DNA fragment was cloned into the expression vector pQE‐30 (Qiagen, Hilden, Germany) to generate the recombinant plasmid pQE‐30‐Clp. The recombinant plasmid pQE‐30‐Clp was then transformed into *E. coli *JM109, resulting in strain JM109/pQE‐30‐Clp. This strain was cultured to an OD_600_ of 0.6, and 1.0 mm of isopropyl‐thiogalactopyranoside (IPTG) was added. After the culture had been grown for a further 4 h, the cells were harvested and the fused protein was purified using Ni‐NTA resin (Qiagen). To obtain the HpaR1 protein, *E. coli* strain JM109/pQE‐30‐2736 expressing HpaR1 with a 6×His tag on its N‐terminus was grown and induced by IPTG (An *et al.*, 2011).

### *In vitro* transcription assay

*In vitro* transcription assays were carried out as described previously (Su *et al.*, 2016). Briefly, the template DNA fragments containing the *engXCA* promoter were generated by PCR amplification from wild‐type strain 8004 with the primer set eng‐ivtF/R (Table S5). After purification using Ni‐NTA resin, 6×His‐tagged HpaR1 or Clp was further dialysed to remove imidazole and incubated with 2 nm of template DNA in transcription buffer for 30 min, followed by the addition of an NTP mixture (250 μm each of ATP, CTP and GTP; 250 μm biotin‐16‐UTP) and 0.1 U of *E. coli *RNAP holoenzyme (sigma‐saturated) to start the transcription. After incubation at 28 °C for 30 min, the reactions were stopped and the transcription products were analysed by electrophoresis.

### EMSA

DNA fragments (1.0 or 2.0 nm) containing the *engXCA *promoter, amplified by PCR using the FAM‐labelled primer sets (P_−184/+47_‐F/R, P_−141/+47_‐F/R, P_−91/+47_‐F/R, P_−56/+47_‐F/R and P_−36/+120_‐F/R, Table S5), were mixed with the purified 6×His‐tagged HpaR1 and/or Clp protein(s) in 20 μL of binding buffer [20 mm Tris‐HCl, 10 mm NaCl, 1 mm ethylenediminetetraacetic acid (EDTA) and 1 mm dithiothreitol, pH 8.0] containing 1 μg of sonicated salmon sperm DNA and 3 μg of bovine serum albumin, and incubated at 30 °C for 20 or 40 min. Samples were then loaded onto a 6% polyacrylamide‐Tris‐borate‐EDTA (TBE) gel, and visualized after electrophoresis.

To quantify the binding affinity of HpaR1 (or Clp) with the *engXCA* promoter, FAM‐labelled DNA fragments (2 nm) were mixed with various amounts of HpaR1 or Clp and incubated at 30 °C for 20 min. After electrophoresis, the gel was scanned with a Bio‐Rad (Hercules, CA, USA) Pharos FXTM Plus scanner, and the data were analysed with Image J software (https://imagej.nih.gov/ij/). The dissociation constant (*K*_d_) was calculated using OriginPro8 software (OriginLab, Northampton, MA, USA).

### Dye primer‐based DNase I footprinting assays

Dye primer‐based DNase I footprinting assays were performed as described previously (Su *et al.*, 2016). In brief, FAM‐labelled DNA fragments containing the *engXCA *promoter were obtained by PCR from the total DNA of *Xcc* strain 8004 using the FAM‐labelled primer sets (Table S5). For investigation of the HpaR1‐protected sequence, a 440‐bp DNA fragment amplified with the primer set Dye‐1F/R was used. For Clp‐protected sequences, 378‐ and 333‐bp fragments amplified with primer sets Dye‐2F/R and Dye‐3F/R, respectively, were used. About 0.5 μm of FAM‐labelled DNA fragments was used for incubation with HpaR1 (10 μm) or Clp (20 or 40 μm) protein. After digestion with DNase I (New England BioLabs), DNA fragments were purified and the digested DNA was added to HiDi formamide (Applied Biosystems) and GeneScan‐500 LIZ size standards (Applied Biosystems). The DNA samples were then analysed with a 3730 DNA Analyzer. The results were analysed with GeneMarker software (Softgenetics).

### Site‐directed mutagenesis

For 13/10‐nucleotide deletions in the HpaR1 and Clp binding sequences (or nucleotide substitution in –35/–10 elements), we first cloned a 303‐bp region of the *engXCA* promoter into the suicide plasmid pK18*mob *(Schäfer *et al.*, 1994) to make a recombinant plasmid pK_engXCA_ *. *For this, we used primer set P_engXCA_‐1F/R (Table S5) to amplify the region with *Eco*RI and *Bam*HI ends to facilitate cloning. Site‐directed mutagenesis was then carried out using a QuikChange™ II Site‐directed Mutagenesis Kit (Stratagene) with the recombinant plasmid pK_engXCA_ as the template and the appropriate primer sets (Table S5). The plasmids with nucleotide deletion or substitution obtained from the site‐directed mutagenesis were verified by sequencing. To obtain FAM‐labelled DNA fragments used for EMSA, the plasmids with nucleotide deletion were used as templates for PCR amplification of the 178/175‐bp (from −141 to +47 relative to TIS) mutant *engXCA* promoter fragments with the FAM‐labelled primer pair P_−141/+47_‐F/R (Table S5). To determine the activity of the mutant *engXCA* promoters, the plasmids with nucleotide substitution were digested with *Eco*RI/*Bam*HI to release the 303‐bp DNA fragments containing the mutant *engXCA* promoters, which were then cloned into plasmid pL6*gus* (Table S4). The resulting recombinant plasmids pGUSengXCA_AT_ and pGUSengXCA_CA_ were introduced by triparental conjugation into *Xcc* strains for GUS activity assay, as described previously (Tang *et al.*, 1991).

To prepare mutant *engXCA* promoter with nucleotide substitution in the ‘−10’ or ‘−35’ element (or 10/13‐nucleotide deletions in the binding sequence) used for *in vitro* transcription assay, a 317‐bp DNA fragment (spanning nucleotides −191 to +126 relative to TIS of the *engXCA* promoter) was PCR amplified with the primer set P_engXCA_‐2F/R. The DNA fragment obtained was cloned into the suicide plasmid pK18*mob *(Schäfer *et al.*, 1994) to make a recombinant plasmid pK_eng317_. Site‐directed mutagenesis was then carried out using the corresponding primer sets (Table S5). The plasmids obtained carrying an *engXCA* promoter with nucleotide substitution were digested with *Eco*RI/*Bam*HI to release the 317‐bp DNA fragments containing the mutant *engXCA* promoters, and the fragments were used as templates for *in vitro* transcription assays.

### ChIP assay

For ChIP assay, a strain producing an HpaR1 protein fused with 3×Flag‐tag (3×Flag::HpaR1) at the N‐terminus of HpaR1, or a Clp protein fused with 3×Flag‐tag (3×Flag::Clp) at the N‐terminus of Clp, was first constructed. A DNA fragment encoding HpaR1 (or Clp) fused with 3×Flag peptide with *Bam*HI‐ and *Hin*dIII‐compatible ends was PCR amplified using the primer set Hflag‐F/R (or Hflag‐F/R) (Table S5). The fragments obtained were cloned into the *Bam*HI/*Hin*dIII sites of the vector pLAFR3, resulting in a recombinant plasmid named pHpa‐Flag (or pClp‐Flag). The plasmid pLAFR3 is a low‐copy‐number vector with an *E. coli lac* promoter flanking the multiple cloning sites, which expresses constitutively in *Xcc* (Huang *et al.*, 2009). The recombinant plasmid pHpa‐Flag (or pClp‐Flag) was introduced into *Xcc* HpaR1 deletion strain ΔHpaR1 (or Clp deletion strain ΔClp) by triparental conjugation, resulting in strain ΔHpaR1/pHpa‐Flag (or ΔClp/pClp‐Flag) (Table S4). As a negative control, the empty vector pLAFR3 was also transformed into *Xcc* wild‐type strain 8004, yielding strain 8004/pLAFR3.

*Xcc* strains were grown in NYG medium for 24 h and cross‐linked by the addition of formaldehyde to a final concentration of 1%. After incubation for 20 min at room temperature with slow shaking, glycine was added at a final concentration of 0.125 m to quench the cross‐linking reaction. Bacterial cells were collected by centrifugation at 8000 ***g*** at 4 °C for 5 min and washed twice in phosphate‐buffered saline (PBS). To lyse the cells, 10 mL of RIPA buffer (50 mm Tris‐HCl, pH 7.4, 150 mm NaCl, 1 mm EDTA, 1% NP‐40, 0.5% sodium deoxycholate) was added and thoroughly mixed by vortexing, and then disrupted by sonication. For each ChIP sample, 50 μL of anti‐Flag (agarose conjugated) were added to the bacterial lysates, and incubated with gentle shaking at 4 °C overnight. Unbound DNA fragments were washed using RIPA buffer, and the bound DNA fragments and proteins were eluted by 0.25 m glycine (pH 2.5).

### GUS activity assay

GUS activity was determined by measurement of *A*_415_ using *p*‐nitrophenyl‐β‐d‐glucuronide as the substrate, as described by Henderson *et al.* (1985), after growth of the *Xcc* strains in medium.

### Transcriptome analysis of the *hpaR1* mutant

To prepare RNA for transcriptome analysis, single bacterial colonies were picked and grown in 5 mL of NYG medium at 28 °C for 24 h at 200 rpm. These cells were transferred into 50 mL of NYG medium again at 28 °C for 24 h at 200 rpm. RNA was harvested once the cell turbidity had reached an OD_600_ of 0.6, using a Total RNA Isolation System (Promega) according to the manufacturer’s protocol. Contaminating genomic DNA was removed using RNase‐free DNase I and verified by PCR. RNA quantity was initially determined by a Nanodrop spectrophotometer ND‐8000 (NanoDrop Technologies, Wilmington, DE, USA), and RNA quality was assessed using an Agilent 2100 bioanalyzer (Agilent Technologies, CA, USA). Total RNA was sent to Novogene (Beijing, China) for library construction and strand‐specific RNA sequencing. Sequencing libraries were generated using a NEBNext Ultra™ Directional RNA Library Prep Kit for Illumina (New England BioLabs) following the manufacturer’s recommendations, and sequenced on an Illumina (CA, USA) HiSeq 2000 platform. Clean reads were mapped to the reference genome. To eliminate the influence of different gene length and sequencing discrepancy on the calculation of gene expression, the RPKM (reads per kilobase per million mapped reads) method was used to calculate the gene expression levels.

## Supporting information


**Fig. S** **1** Electrophoretic mobility shift assays (EMSAs) for narrowing down the binding sites of HpaR1 and Clp proteins on the *engXCA* promoter of *Xanthomonas campestris* pv. *campestris* (*Xcc*). (A) Polymerase chain reaction (PCR) fragments in series length used as probes in EMSAs. Horizontal lines represent the DNA fragments corresponding to the region within the *engXCA* promoter, the names of the probes are indicated on the left and the numbers after P are the nucleotide positions relative to the transcription initiation site (TIS) of *engXCA*. Primers used for PCR amplification are listed in Table S5. (B) EMSAs to test the binding of HpaR1 with a variety of probes; 1.0 nm DNA fragments were incubated with 0, 10 and 20 nm of HpaR1 proteins at 30 ºC for 20 min. (C) EMSAs to test the binding of Clp with a variety of probes; 1.0 nm DNA fragments were incubated with 0, 20 and 30 nm of Clp proteins at 30 ºC for 20 min.

**Fig. S2** Nucleotide substitution in −35 and −10 elements reduces *engXCA* promoter activity. (A) The β‐glucuronidase (GUS) activity of *engXCA *promoter‐*gusA *reporters in the wild‐type strain 8004. Two *engXCA* mutant promoters [spanning nucleotides −256 to +47 relative to the transcription initiation site (TIS)] with ‘CAAGAT’ and ‘ATCACA’ shifted to ‘CAAGCG’ and ‘ATCAGT’, respectively, were created by site‐directed mutagenesis (see Experimental procedures). These two mutant promoters were fused to the promoterless *gusA *gene, resulting in reporter plasmids pGUSengXCA_AT_ and pGUSengXCA_CA_, respectively. GUS activity was measured after the bacterial cells had been cultured in NYG medium containing 2% glucose for 24 h. The values given are the means ± standard deviations of triplicate measurements from a representative experiment; similar results were obtained in two other independent experiments. (B) *In vitro *transcription assay. Two *engXCA* mutant promoters (spanning nucleotides −191 to +126 relative to TIS) with nucleotide substitution in the −10 and −35 elements were first created (see Experimental procedures); 2 nm DNA of the 317‐bp fragments of the wild‐type *engXCA *promoter, or mutants of the *engXCA *promoter, were incubated with 0.1 U of RNA polymerase (RNAP). Transcription products were then run on a 5% denatured polyacrylamide gel containing 7 m urea in 1 × Tris‐borate‐EDTA (TBE) electrophoresis buffer.

**Fig. S3** Mutation analysis of the HpaR1 and Clp binding sites on the *engXCA *promoter. (A) Electrophoretic mobility shift assays (EMSAs) of the mutated promoter; 13 and 10 nucleotides within HBS/CBS II and CBS I were chosen for deletion by site‐directed mutagenesis (see Experimental procedures). The resulting mutant fragments were designated as HBS‐13 and CBS I‐10. 6‐Carboxyfluorescein (FAM)‐labelled *engXCA* promoter DNA fragments [spanning nucleotides −141 to +47 relative to the transcription initiation site (TIS) of the *engXCA *promoter] with or without (wild‐type) mutations were incubated with increasing amounts of HpaR1 (i) or Clp (ii, iii) protein for 40 min at 30 ºC before EMSA. (B) *In vitro *transcription assays of the mutated promoter. DNA fragments (spanning nucleotides −191 to +126 relative to TIS of the *engXCA* promoter) with 13‐ and 10‐nucleotide deletions within HBS/CBS II and CBS I, respectively, were constructed (see Experimental procedures). The resulting DNA fragments P_engXCA/HBS_ or P_engXCA/CBS I_, and the wild‐type fragment P_engXCA_, were subjected to *in vitro *transcription assays with amounts of HpaR1 (i) or Clp (ii, iii) protein.

**Table S** **1** Gene expression profile of the ΔhpaR1 strain when grown in NYG. Note: false discovery rate (FDR) = 0.05 and absolute value of log_2_ of the fold change (log_2_FC) = 1 (equivalent to a fold change of two) were used as the cut‐off values. ‘+’ represents genes up‐regulated in the mutant ΔhpaR1 and ‘−’ represents genes down‐regulated.

**Table** **S2** Confirmation of RNA sequencing (RNA‐seq) gene expression data by semi‐quantitative real‐time polymerase chain reaction (RT‐PCR).

**Table S3** Genes regulated by both global transcriptional regulators HpaR1 and Clp.

**Table** **S4** Bacterial strains and plasmids used in this work. ^a^Rif^r^, Kan^r^, Tet^r^ and Spc^r^ indicate resistance to rifampicin, kanamycin, tetracycline and spectinomycin, respectively.

**Table S** **5** Primers used in this study.
