# Supplementary figures and images for: Competitive control of endoglucanase gene engXCA expression in the plant pathogen Xanthomonas campestris by the global transcriptional regulators HpaR1 and Clp
Source: Mol Plant Pathol. 2018 Oct 9;20(1):51–68. doi: 10.1111/mpp.12739 (PMC6430473; doi:10.1111/mpp.12739)

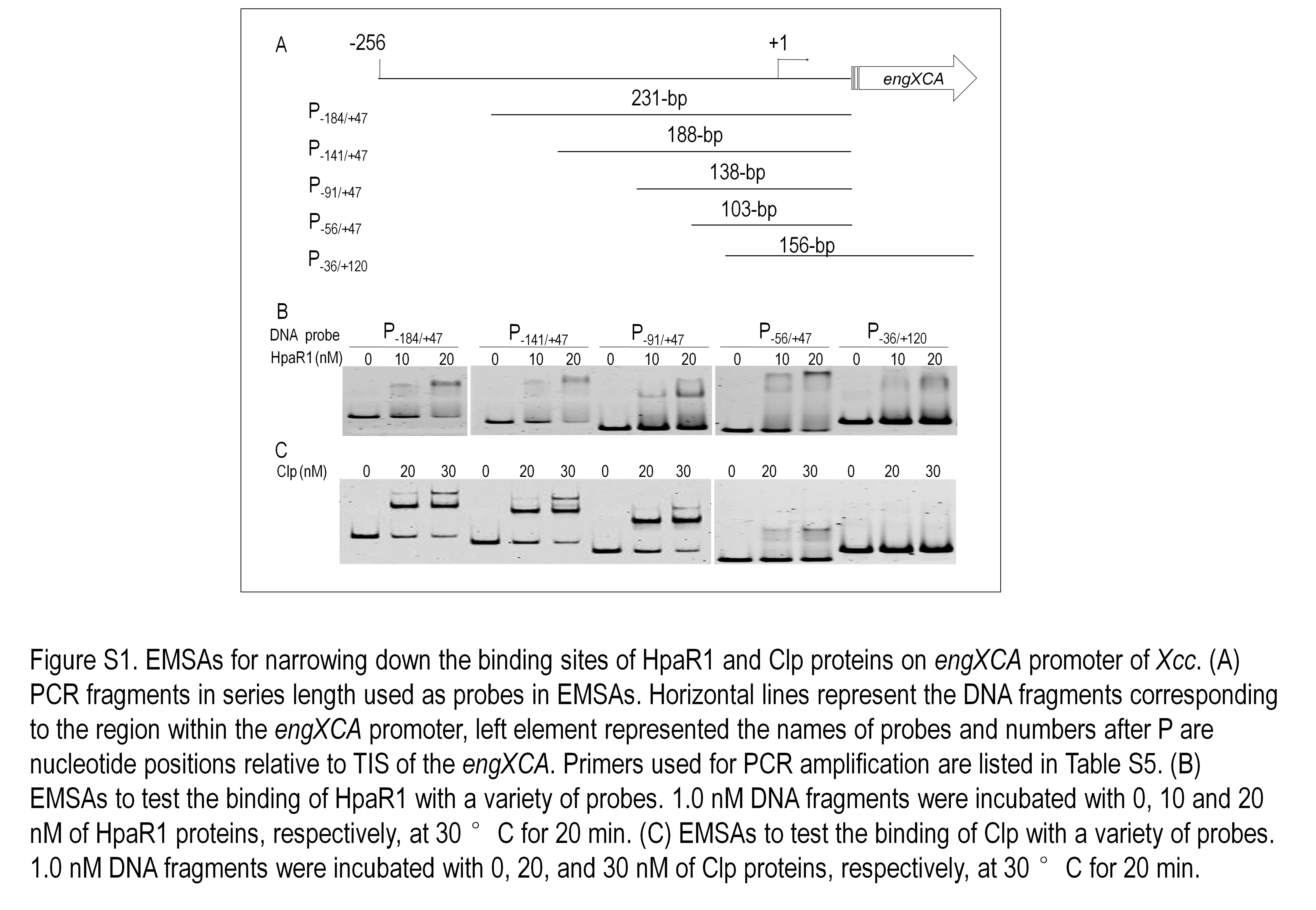

Supplement: Supplementary file 1 — Fig. S 1 Electrophoretic mobility shift assays (EMSAs) for narrowing down the binding sites of HpaR1 and Clp proteins on the engXCA promoter of Xanthomonas campestris pv. campestris (Xcc). (A) Polymerase chain reaction (PCR) fragments in series length used as probes in EMSAs. Horizontal lines represent the DNA fragments corresponding to the region within the engXCA promoter, the names of the probes are indicated on the left and the numbers after P are the nucleotide positions relative to the transcription initiation site (TIS) of engXCA. Primers used for PCR amplification are listed in Table S5. (B) EMSAs to test the binding of HpaR1 with a variety of probes; 1.0 nm DNA fragments were incubated with 0, 10 and 20 nm of HpaR1 proteins at 30 ºC for 20 min. (C) EMSAs to test the binding of Clp with a variety of probes; 1.0 nm DNA fragments were incubated with 0, 20 and 30 nm of Clp proteins at 30 ºC for 20 min. [file MPP-20-51-s001.jpg]

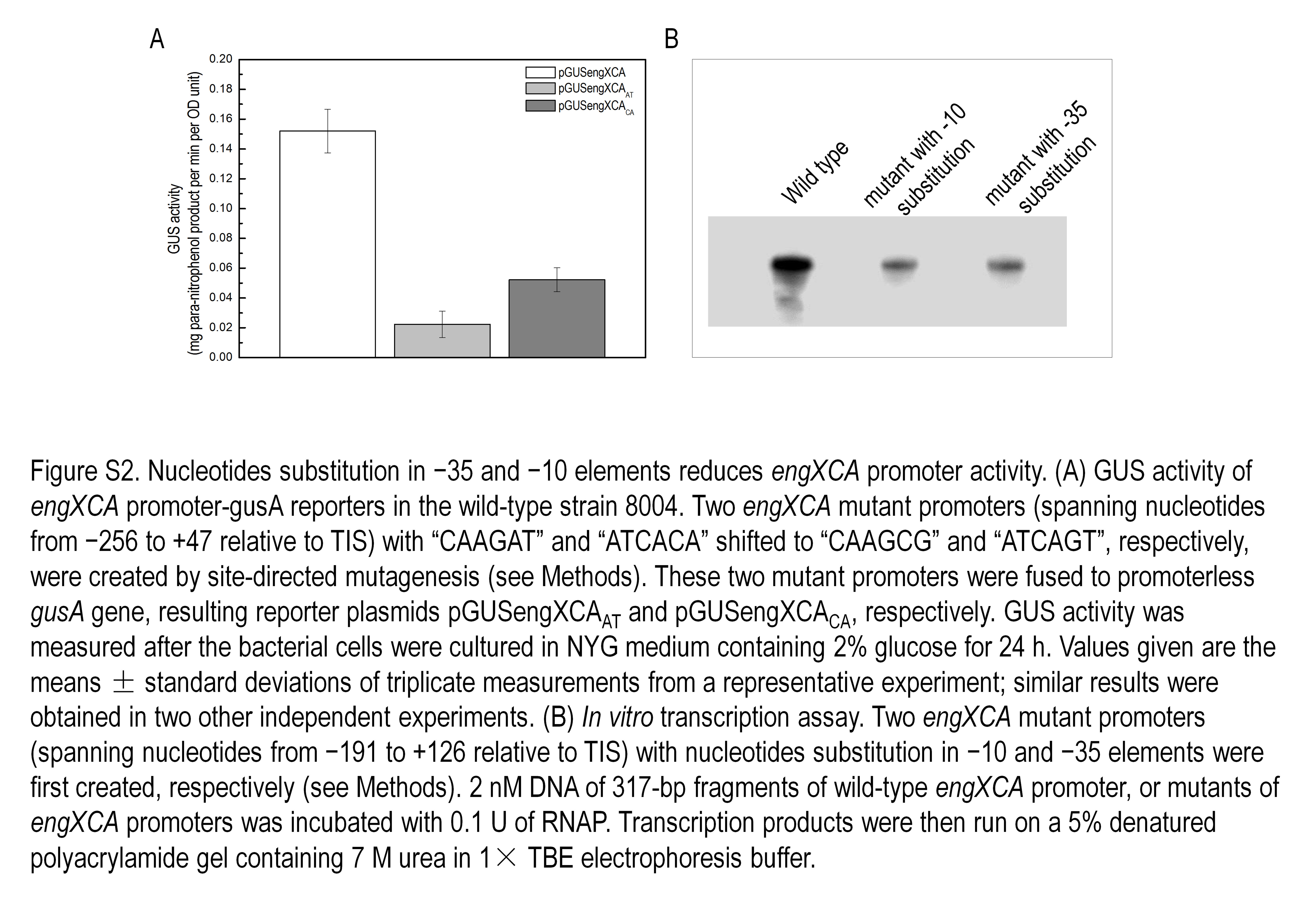

Supplement: Supplementary file 2 — Fig. S2 Nucleotide substitution in −35 and −10 elements reduces engXCA promoter activity. (A) The β‐glucuronidase (GUS) activity of engXCA promoter‐gusA reporters in the wild‐type strain 8004. Two engXCA mutant promoters [spanning nucleotides −256 to +47 relative to the transcription initiation site (TIS)] with ‘CAAGAT’ and ‘ATCACA’ shifted to ‘CAAGCG’ and ‘ATCAGT’, respectively, were created by site‐directed mutagenesis (see Experimental procedures). These two mutant promoters were fused to the promoterless gusA gene, resulting in reporter plasmids pGUSengXCAAT and pGUSengXCACA, respectively. GUS activity was measured after the bacterial cells had been cultured in NYG medium containing 2% glucose for 24 h. The values given are the means ± standard deviations of triplicate measurements from a representative experiment; similar results were obtained in two other independent experiments. (B) In vitro transcription assay. Two engXCA mutant promoters (spanning nucleotides −191 to +126 relative to TIS) with nucleotide substitution in the −10 and −35 elements were first created (see Experimental procedures); 2 nm DNA of the 317‐bp fragments of the wild‐type engXCA promoter, or mutants of the engXCA promoter, were incubated with 0.1 U of RNA polymerase (RNAP). Transcription products were then run on a 5% denatured polyacrylamide gel containing 7 m urea in 1 × Tris‐borate‐EDTA (TBE) electrophoresis buffer. [file MPP-20-51-s002.jpg]

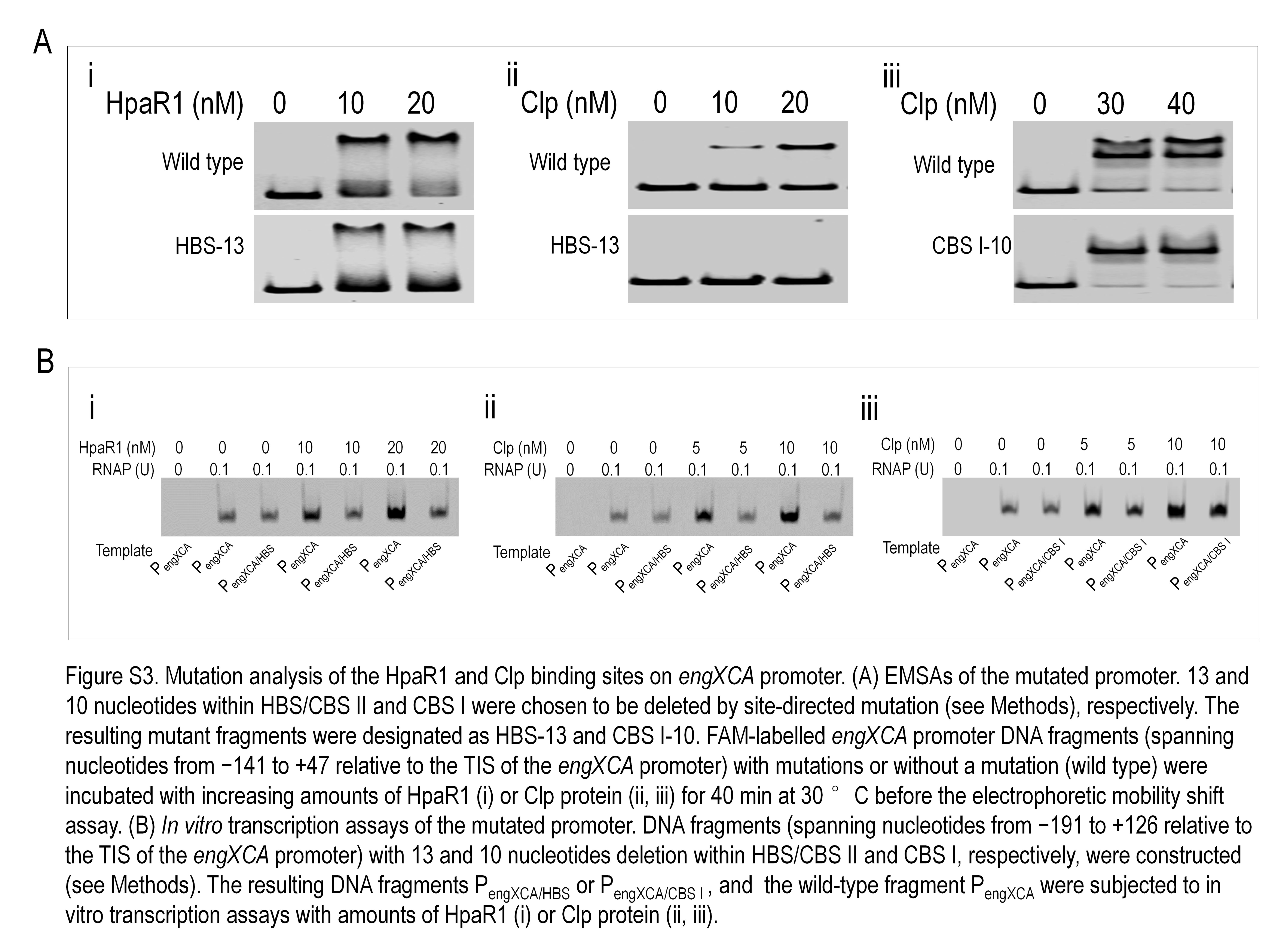

Supplement: Supplementary file 3 — Fig. S3 Mutation analysis of the HpaR1 and Clp binding sites on the engXCA promoter. (A) Electrophoretic mobility shift assays (EMSAs) of the mutated promoter; 13 and 10 nucleotides within HBS/CBS II and CBS I were chosen for deletion by site‐directed mutagenesis (see Experimental procedures). The resulting mutant fragments were designated as HBS‐13 and CBS I‐10. 6‐Carboxyfluorescein (FAM)‐labelled engXCA promoter DNA fragments [spanning nucleotides −141 to +47 relative to the transcription initiation site (TIS) of the engXCA promoter] with or without (wild‐type) mutations were incubated with increasing amounts of HpaR1 (i) or Clp (ii, iii) protein for 40 min at 30 ºC before EMSA. (B) In vitro transcription assays of the mutated promoter. DNA fragments (spanning nucleotides −191 to +126 relative to TIS of the engXCA promoter) with 13‐ and 10‐nucleotide deletions within HBS/CBS II and CBS I, respectively, were constructed (see Experimental procedures). The resulting DNA fragments PengXCA/HBS or PengXCA/CBS I, and the wild‐type fragment PengXCA, were subjected to in vitro transcription assays with amounts of HpaR1 (i) or Clp (ii, iii) protein. [file MPP-20-51-s003.jpg]
