# Supplementary material for: Competitive control of endoglucanase gene engXCA expression in the plant pathogen Xanthomonas campestris by the global transcriptional regulators HpaR1 and Clp
Source: Mol Plant Pathol. 2018 Oct 9;20(1):51–68. doi: 10.1111/mpp.12739 (PMC6430473; doi:10.1111/mpp.12739)
Supplement: Supplementary file 4 — Table S 1 Gene expression profile of the ΔhpaR1 strain when grown in NYG. Note: false discovery rate (FDR) = 0.05 and absolute value of log2 of the fold change (log2FC) = 1 (equivalent to a fold change of two) were used as the cut‐off values. ‘+’ represents genes up‐regulated in the mutant ΔhpaR1 and ‘−’ represents genes down‐regulated. [file MPP-20-51-s004.docx]

**Table S1.** The up- and down-regulated differential expressed genes of the *hpaR1* mutant strain cultured in the nutrition medium NYG.

| Functional category | ORF number | Gene name | Predicted product | Expression fold change *(*△hpaR1/wt) |
| --- | --- | --- | --- | --- |
| Amino acids biosynthesis | XC_1103  XC_0982  XC_0983  XC_1090  XC_2177 | *lysA*  *cysK*  *cysG*  *metB*  *nasD* | diaminopimelate decarboxylase  cysteine synthase  siroheme synthase  homocysteine synthase  nitrite reductase [NAD(P)H] | 2.51  -4.42  -4.81  -3.29  -2.04 |
| Biosynthesis of cofactors, prosthetic groups, carriers | XC_3952  XC_2471 | *entF* | ATP-dependent serine activating enzyme  GTP cyclohydrolase | -2.79  -2.47 |
| Cell envelope and cell structure | XC_1459  XC_2862  XC_0894 | *phuR*  *oar* | outer membrane hemin receptor  pili assembly chaperone  Oar protein | 2.11  3.12  -2.59 |
| Cellular processes | XC_0286  XC_0638  XC_1074  XC_1409  XC_1410  XC_1412  XC_1413  XC_1414  XC_1801  XC_2223  XC_2231  XC_2233  XC_2234  XC_2235  XC_2236  XC_2237  XC_2238  XC_2239  XC_2240  XC_2241  XC_2242  XC_2243  XC_2244  XC_2245  XC_2246  XC_2247  XC_2259  XC_2260  XC_2261  XC_2263  XC_2264  XC_2265  XC_2266  XC_2267  XC_2268  XC_2269  XC_2270  XC_2277  XC_2278  XC_2279  XC_2280  XC_2282  XC_2283  XC_2284  XC_2297  XC_2298  XC_2299  XC_2300  XC_2302  XC_2303  XC_2306  XC_2307  XC_2308  XC_2309  XC_2311  XC_2313  XC_2315  XC_2316  XC_2318  XC_2320  XC_2321  XC_2322  XC_2323  XC_2504  XC_3724  XC_3725 | *tsr*  *tsr*  *mcp1*  *cheB*  *cheR*  *cheW*  *mcp*  *cheA*  *mcp*  *mcp*  *flgM*  *cheV*  *flgB*  *flgC*  *flgD*  *flgE*  *flgF*  *flgG*  *flgH*  *flgI*  *flgJ*  *flgK*  *flgL*  *fliC*  *fliD*  *fliS*  *fliE*  *fliF*  *fliG*  *fliI*  *fliJ*  *fliK*  *fliL*  *fliM*  *fliN*  *fliO*  *fliP*  *flhB*  *flhA*  *flhF*  *fleN*  *cheY*  *cheZ*  *cheA*  *motA*  *motB*  *parA*  *cheW*  *cheY*  *cheA*  *tsr*  *tsr*  *tsr*  *tsr*  *tsr*  *tsr*    *tsr*  *cheW*  *tsr*  *cheR*  *cheD*  *cheB*  *mcpA*  *motA*  *motB* | chemotaxis protein  chemotaxis protein  chemotaxis protein  chemotaxis-specific methylesterase  response regulator for chemotaxis  chemotaxis protein  chemotaxis protein  chemotaxis histidine protein kinase  chemotaxis protein  chemotaxis protein  flagellar protein  chemotaxis protein  flagellar basal body rod protein FlgB  flagellar basal body rod protein FlgC  flagellar basal body rod modification protein  flagellar hook protein FlgE  flagellar basal body rod protein FlgF  flagellar basal body rod protein FlgG  flagellar basal body L-ring protein  flagellar basal body P-ring protein  flagellar rod assembly protein/muramidase FlgJ  flagellar hook-associated protein FlgK  flagellar hook-associated protein FlgL  flagellin  flagellar protein  flagellar protein  flagellar protein  flagellar MS-ring protein  flagellar protein  flagellar protein  flagellar FliJ protein  flagellar protein  flagellar biosynthesis protein  flagellar motor switch protein FliM  flagellar protein  flagellar protein  flagellar biosynthesis protein FliP  flagellar biosynthesis protein FlhB  flagellar biosynthesis protein FlhA  flagellar biosynthesis regulator FlhF  flagellar biosynthesis switch protein  chemotaxis protein  chemotaxis related protein  chemotaxis related protein  flagellar motor protein  flagellar motor protein MotD  chromosome partioning protein  chemotaxis protein  chemotaxis response regulator  chemotaxis protein  chemotaxis protein  chemotaxis protein  chemotaxis protein  chemotaxis protein  chemotaxis protein  chemotaxis protein  methyl-accepting chemotaxis protein  chemotaxis protein  chemotaxis protein  chemotaxis protein  chemotaxis protein methyltransferase  chemoreceptor glutamine deamidase CheD  chemotaxis-specific methylesterase  chemotaxis protein  flagellar motor protein MotA  flagellar motor protein MotB | 2.73  3.60  2.72  2.56  3.26  3.05  4.01  2.63  2.21  3.82  3.05  3.07  3.08  2.15  2.44  2.18  2.19  2.27  2.05  2.15  2.31  2.30  2.36  3.57  3.11  2.38  5.67  2.39  2.25  2.30  2.28  2.05  4.22  2.73  2.02  2.18  2.24  2.13  2.33  2.53  2.93  3.18  2.89  2.89  3.09  3.66  3.22  2.90  3.05  3.09  6.23  3.48  2.54  3.34  4.48  3.77  2.37  3.03  2.70  3.75  3.62  2.70  3.28  4.09  2.23  2.26 |
| Central intermediary metabolism | XC_1881  XC_0990  XC_0991  XC_0992  XC_0993  XC_0994  XC_1232  XC_2981  XC_3032  XC_3054  XC_3215  XC_3456  XC_3658 | *gaa*  *cysH*  *cysI*  *cysJ*  *cysD*  *cysN*  *gcvT*  *mmsA*  *lamA*  *prpB*  *tauD*  *gloA* | glutaryl-7-ACA acylase precursor  phosphoadenosine phosphosulfate reductase  sulfite reductase subunit beta  NADPH-sulfite reductase flavoprotein subunit  sulfate adenylyltransferase subunit 2  sulfate adenylyltransferase subunit 1  glycine cleavage system aminomethyltransferase T  methylmalonate-semialdehyde dehydrogenase  tryptophan 2,3-dioxygenase  endo-1,3-beta-glucanase  2-methylisocitrate lyase  taurine dioxygenase  lactoylglutathione lyase | 2.89  -7.22  -6.31  -5.46  -7.47  -7.20  -2.01  -2.30  -2.42  -2.21  -2.13  -5.41  -2.22 |
| Energy and carbon metabolism | XC_0328  XC_0279  XC_0280  XC_0281  XC_0887  XC_0979  XC_3156  XC_3677  XC_3683 | *sflA*  *mocA*  *atpC*  *atpE* | NADH-dependent FMN reductase  2,5-diketo-D-gluconate reductase B  quinone oxidoreductase  oxidoreductase  cytochrome C6  fructose-bisphosphate aldolase  ferredoxin  F0F1 ATP synthase subunit epsilon  F0F1 ATP synthase subunit C | 2.37  -2.63  -2.50  -2.80  -2.13  -2.00  -2.43  -2.18  -2.56 |
| Fatty acid and phospholipid meatbolism | XC_0276  XC_1408  XC_1682 | *cls*  *blc* | lipase  cardiolipin synthase  outer membrane lipoprotein | 3.34  -2.03  -2.56 |
| Nucleotides metabolism |  |  |  |  |
| Regulatory functions | XC_1766  XC_3399  XC_0923 | *rrpX*  *rnk* | transcriptional regulator  regulator of nucleoside diphosphate kinase  transcriptional regulator | 3.63  -2.03  -2.30 |
| Replication and DNA metabolism | XC_2785  XC_1399 |  | helicase  pirin | 3.35  -2.42 |
| Transport | XC_0167  XC_0218  XC_0642  XC_1104  XC_1341  XC_1970  XC_1971  XC_1972  XC_2737  XC_2846  XC_3463  XC_4000  XC_0820  XC_0849  XC_1087  XC_1113  XC_1115  XC_2844  XC_3201  XC_3292  XC_3293  XC_3294  XC_3457  XC_3458  XC_3459  XC_4126 | *fpvA*  *fpvA*  *iucA*  *fhuA*  *czcB*  *acrD*  *acrD*  *nodI*  *fhuA*  *phuR*  *tptC*  *dctA*  *btuB*  *bfeA*  *bfeA*  *brf*  *cysA*  *cysW*  *cysU*  *nrtCD*  *nrtB*  *czcB* | ferripyoverdine receptor  MFS transporter  ferripyoverdine receptor  iron transporter  TonB-dependent receptor  cation efflux system protein  transport protein  transport protein  ABC transporter ATP-binding protein  iron receptor  outer membrane hemin receptor  ABC transporter ATP-binding protein  C4-dicarboxylate transporter DctA  TonB-dependent receptor  ABC transporter ATP-binding subunit  ferric enterobactin receptor  ferric enterobactin receptor  bacterioferritin  bacterioferritin  sulfate ABC transporter ATP-binding protein  sulfate ABC transporter sulfate permease  sulfate ABC transporter sulfate permease  ABC transporter substrate binding protein  ABC transporter ATP-binding component  permease  cation efflux system protein | 5.09  32.31  2.21  3.87  2.16  292.13  221.62  13.96  15.02  2.81  4.18  2.01  -2.86  -4.13  -2.11  -2.15  -7.17  -2.07  -4.16  -2.46  -2.34  -2.27  -6.26  -8.02  -4.12  -2.01 |
| Translation | XC_0094  XC_0096  XC_0219  XC_0240  XC_0253  XC_0611  XC_0636  XC_0643  XC_0654  XC_0997  XC_0998  XC_1007  XC_1544  XC_2538  XC_2826  XC_3192  XC_0667  XC_1291  XC_1292  XC_3280  XC_4390 | *tldD*  *tldD*    *dcp*            *pepN*          *dcp*  *pepN*  *hslV* | TldD protein  TldD protein  tRNA/rRNA methyltransferase  peptidyl-dipeptidase  dipeptidyl anminopeptidase  dipeptidyl peptidase IV  zinc protease  aminopeptidase  prolyl oligopeptidase  aminopeptidase N  acetyltransferase  aminopeptidase  metallopeptidase  carboxypeptidase-related protein  peptidyl-dipeptidase  aminopeptidase N  ATP-dependent protease peptidase subunit  endoproteinase Arg-C  endoproteinase Arg-C  peptidyl-Asp metalloendopeptidase  23S ribosomal RNA | 2.32  2.53  31.55  2.54  5.61  2.36  2.35  2.84  2.37  3.69  3.37  2.38  4.56  2.82  3.48  3.16  -2.34  -2.72  -3.47  -2.43  -4.03 |
| Transcription | XC_2281  XC_2934  XC_2251 | *fliA*  *algU*  *rpoN* | RNA polymerase sigma factor  RNA polymerase sigma factor  RNA polymerase sigma-54 factor | 2.63  2.21  2.20 |
| Signal transduction | XC_3117  XC_3118  XC_2275  XC_0637  XC_0420  XC_1476  XC_2276  XC_0641  XC_227 |  | two-component system regulatory protein  two-component system sensor protein  GGDEF family protein  histidine kinase/response regulator hybrid protein  GGDEF family protein  GGDEF family protein  GGDEF family protein  GGDEF family protein  GGDEF family protein | 3.12  2.25  2.99  2.97  2.52  2.25  2.25  2.12  2.11 |
| Mobile genetic elements | XC_2438  XC_0702  XC_3942  XC_3622  XC_3919  XC_2083 | *IS1478*  *IS1478* | plasmid-related protein  IS1478 transposase  IS1478 transposase  Transposase  Transposase  transposase | -2.25  -2.45  -2.63  -2.92  -2.07  -2.37 |
| Pathogenicity and adaptation | XC_1411  XC_1811  XC_2324  XC_3376  XC_3860  XC_3861  XC_0026  XC_0027  XC_0639  XC_0160  XC_0741  XC_1005  XC_1027  XC_1298  XC_1515  XC_1658  XC_1664  XC_1667  XC_1668  XC_1669  XC_2558  XC_2735  XC_3200  XC_3590  XC_3591  XC_4010  XC_4318 | *vieA*  *acvB*  *pdeA*  *acrD*  *acrA*  *egl*  *egl*  *engXCA*  *kduI*  *xcsF*  *virB6*  *pelB*  *gumB*  *gumH*  *gumK*  *gumL*  *gumM*  *btuE*  *pel*  *pel*  *avrXccA1* | response regulator  virulence protein  c-di-GMP phosphodiesterase A  extracellular protease  acriflavin resistance protein  acriflavin resistance protein  cellulase  cellulase  major extracellular endoglucanase  5-keto-4-deoxyuronate isomerase  type II secretion system protein F  1,4-beta-cellobiosidase  VirB6 protein  pectate lyase II  extracellular protease  GumB protein  GumH protein  GumK protein  GumL protein  GumM protein  glycosyl transferase  ABC transporter permease vitamin B12  peroxiredoxin  pectate lyase  pectate lyase  beta-lactamase related protein  avirulence protein | 3.38  2.04  2.96  2.17  2.32  3.21  -2.01  -4.53  -3.04  -2.28  -2.07  -3.14  -2.05  -3.59  -2.53  -2.00  -2.06  -2.59  -2.98  -3.01  -2.03  -2.91  -2.63  -2.67  -16.11  -2.08  -2.55 |
| Undefined category | XC_1201  XC_2147  XC_4362  XC_2843  XC_395 | *rebB*  *tpmT*  *ugt* | RebB protein  Hydroxylase  transfer-messenger RNA  thiopurine S-methyltransferase  glucosyltransferase | 3.08  3.03  3.45  -2.38  -2.61 |
| Hypothetical proteins | XC_0220  XC_0023  XC_0093  XC_0169  XC_0217  XC_0251  XC_0252  XC_0263  XC_0329  XC_0347  XC_0362  XC_0656  XC_0657  XC_1106  XC_1107  XC_1127  XC_1202  XC_1339  XC_1369  XC_1415  XC_1485  XC_1708  XC_1709  XC_1710  XC_2142  XC_2154  XC_2165  XC_2224  XC_2226  XC_2230  XC_2248  XC_2249  XC_2301  XC_2305  XC_2312  XC_2317  XC_2319  XC_2459  XC_2539  XC_2738  XC_2739  XC_2740  XC_2741  XC_2786  XC_2787  XC_2788  XC_2795  XC_2830  XC_2836  XC_2861  XC_3080  XC_3128  XC_3191  XC_3367  XC_3461  XC_3462  XC_3464  XC_3481  XC_3645  XC_3692  XC_3862  XC_3863  XC_4001  XC_4034  XC_4035  XC_4046  XC_4047  XC_4129  XC_4268  XC_1400  XC_3798  XC_0230  XC_0508  XC_0619  XC_0786  XC_0787  XC_0795  XC_0880  XC_0903  XC_1114  XC_1159  XC_1353  XC_1559  XC_1718  XC_2420  XC_2436  XC_2437  XC_2582  XC_2963  XC_3203  XC_3206  XC_3540  XC_3675  XC_3686  XC_3911  XC_3912  XC_3956  XC_4012  XC_4308 |  | hypothetical protein  hypothetical protein  hypothetical protein  hypothetical protein  hypothetical protein  hypothetical protein  hypothetical protein  hypothetical protein  hypothetical protein  hypothetical protein  hypothetical protein  hypothetical protein  hypothetical protein  hypothetical protein  hypothetical protein  hypothetical protein  hypothetical protein  hypothetical protein  hypothetical protein  hypothetical protein  hypothetical protein  hypothetical protein  hypothetical protein  hypothetical protein  hypothetical protein  hypothetical protein  hypothetical protein  hypothetical protein  hypothetical protein  hypothetical protein  hypothetical protein  hypothetical protein  hypothetical protein  hypothetical protein  hypothetical protein  hypothetical protein  hypothetical protein  hypothetical protein  hypothetical protein  hypothetical protein  hypothetical protein  hypothetical protein  hypothetical protein  hypothetical protein  hypothetical protein  hypothetical protein  hypothetical protein  hypothetical protein  hypothetical protein  hypothetical protein  hypothetical protein  hypothetical protein  hypothetical protein  hypothetical protein  hypothetical protein  hypothetical protein  hypothetical protein  hypothetical protein  hypothetical protein  hypothetical protein  hypothetical protein  hypothetical protein  hypothetical protein  hypothetical protein  hypothetical protein  hypothetical protein  hypothetical protein  hypothetical protein  hypothetical protein  hypothetical protein  hypothetical protein  hypothetical protein  hypothetical protein  hypothetical protein  hypothetical protein  hypothetical protein  hypothetical protein  hypothetical protein  hypothetical protein  hypothetical protein  hypothetical protein  hypothetical protein  hypothetical protein  hypothetical protein  hypothetical protein  hypothetical protein  hypothetical protein  hypothetical protein  hypothetical protein  hypothetical protein  hypothetical protein  hypothetical protein  hypothetical protein  hypothetical protein  hypothetical protein  hypothetical protein  hypothetical protein  hypothetical protein  hypothetical protein | 4177.12  5.07  2.12  2.31  6.38  2.66  2.49  2.56  2.09  2.16  3.38  4.09  2.79  4.09  3.00  2.04  2.33  2.07  2.51  2.22  2.30  2.05  4.61  3.22  2.06  2.99  4.40  3.31  2.61  2.98  2.29  2.58  3.54  5.24  3.97  2.78  2.72  2.13  2.14  13.33  14.97  13.84  2.32  4.00  3.87  3.06  2.43  3.51  3.07  4.97  2.49  4.82  2.74  3.87  3.52  2.71  4.73  2.84  5.09  4.59  2.69  2.60  2.13  3.08  2.97  2.13  2.35  2.72  3.20  -2.00  -2.01  -2.10  -2.13  -2.15  -3.45  -3.77  -2.11  -2.41  -2.35  -2.03  -2.12  -2.34  -2.02  -2.75  -2.21  -2.35  -2.08  -2.49  -2.18  -2.52  -2.65  -2.23  -2.05  -2.48  -2.47  -2.29  -2.26  -2.03  -2.19 |

Note: False discovery rate (FDR) =0.05 and absolute value of log_2_FC (log_2_ of the fold change) =1 (equivalent to a fold change of 2) were used as the cut off values. “+” represents genes up-regulated in the mutant ΔhpaR1, and “-” represents genes down-regulated.
