# Supplementary material for: Competitive control of endoglucanase gene engXCA expression in the plant pathogen Xanthomonas campestris by the global transcriptional regulators HpaR1 and Clp
Source: Mol Plant Pathol. 2018 Oct 9;20(1):51–68. doi: 10.1111/mpp.12739 (PMC6430473; doi:10.1111/mpp.12739)
Supplement: Supplementary file 5 — Table S2 Confirmation of RNA sequencing (RNA‐seq) gene expression data by semi‐quantitative real‐time polymerase chain reaction (RT‐PCR). [file MPP-20-51-s005.docx]

**Supplementary Table S2.** Confirmation of RNA-Seq gene expression data by semi-quantitative RT-PCR.

| ID | Gene | Description | Expresstion level | RT-PCR  △hpaR1/wt |
| --- | --- | --- | --- | --- |
| *XC_0251* |  | hypothetical protein | 2.66↑ | 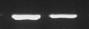 |
| *XC_0286* | *tsr* | chemotaxis protein | 2.73↑ | 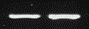 |
| *XC_0420* |  | GGDEF family protein | 2.52↑ | 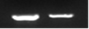 |
| *XC_0508* |  | hypothetical protein | 2.13↓ | 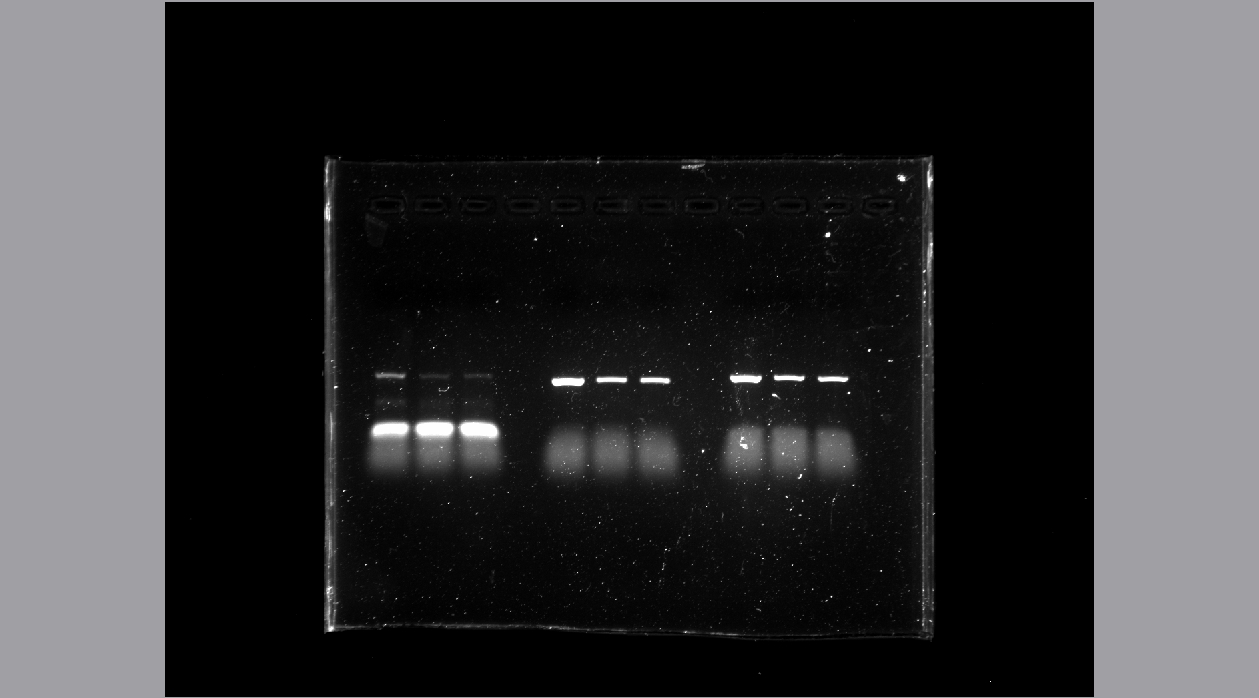 |
| *XC_0992* | *cysJ* | NADPH-sulfite reductase flavoprotein subunit | 5.46 ↓ | 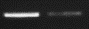 |
| *XC_1005* |  | 1,4-beta-cellobiosidase | 3.14↓ | 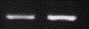 |
| *XC_1087* | *ssuC* | ABC transporter ATP-binding subunit | 2.11↓ | 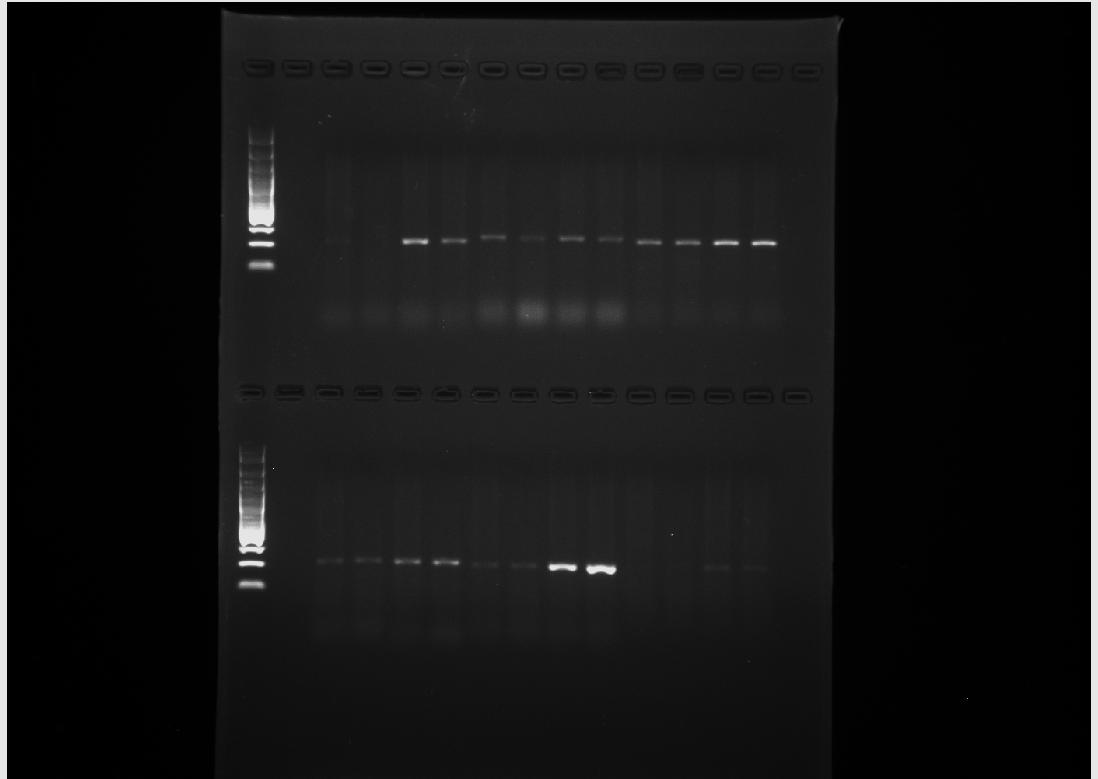 |
| *XC_1292* |  | endoproteinase Arg-C | 3.47↓ | 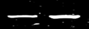 |
| *XC_1408* |  | cardiolipin synthase | 2.03↓ | 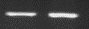 |
| *XC_1544* |  | metallopeptidase | 4.56↑ | 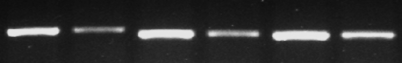 |
| *XC_1658* | *gumB* | GumB protein | 2.00↓ | 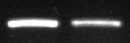 |
| *XC_1972* | *acrD* | transport protein | 13.9↑ | 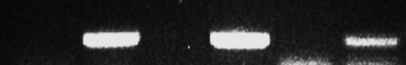 |
| *XC_2235* | *flgC* | flagellar biosynthesis protein | 2.15 ↑ | 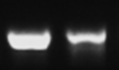 |
| *XC_2239* | *flgG* | flagellar biosynthesis protein | 2.27 ↑ | 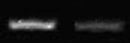 |
| *XC_2277* | *flhB* | flagellar protein | 2.13 ↑ | 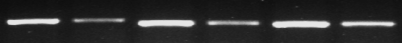 |
| *XC_3117* |  | two-component system regulatory protein | 3.12↑ | 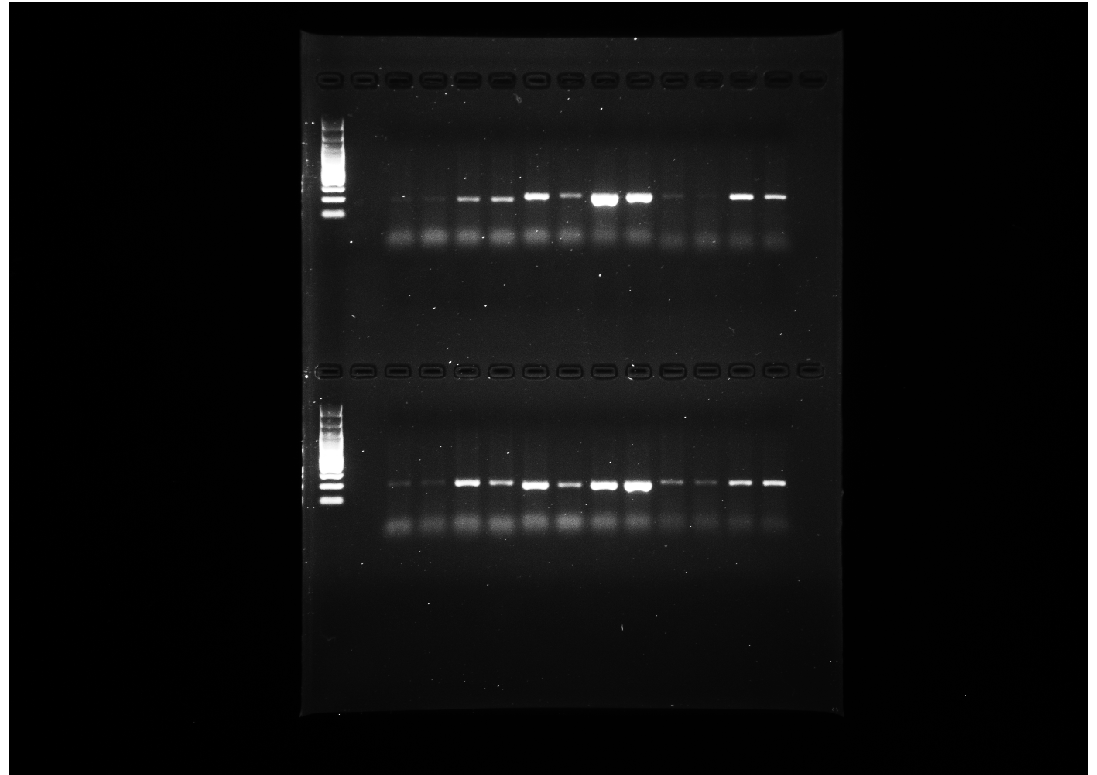 |
| *XC_3128* |  | conserved hypothetical protein | 4.82 ↑ | 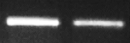 |
| *XC_3201* |  | bacterioferritin | 4.16↓ | 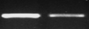 |
| *XC_3456* | *tauD* | Central intermediary metabolism | 5.41 ↓ | 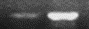 |
| *XC_4010* |  | beta-lactamase related protein | 2.08↓ | 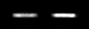 |
| 16S |  |  |  | 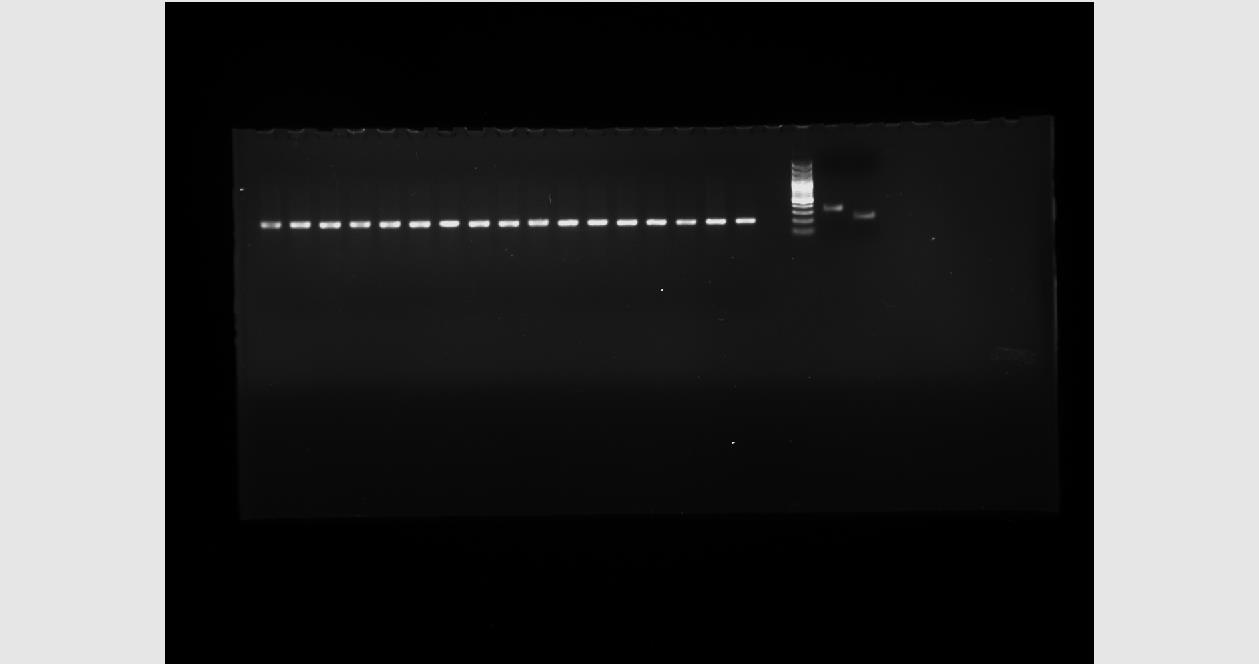 |

**Note: Validation of differentially expressed genes by semi quantitative reverse-transcription PCR (semi RT-PCR).** In this study, false discovery rate (FDR) =0.05 and absolute value of log_2_foldchange =1 (equivalent to a fold change of 2) were used as the cut off values. ↑: up-regulated; ↓: down-regulated.
