# Supplementary material for: Competitive control of endoglucanase gene engXCA expression in the plant pathogen Xanthomonas campestris by the global transcriptional regulators HpaR1 and Clp
Source: Mol Plant Pathol. 2018 Oct 9;20(1):51–68. doi: 10.1111/mpp.12739 (PMC6430473; doi:10.1111/mpp.12739)
Supplement: Supplementary file 6 — Table S3 Genes regulated by both global transcriptional regulators HpaR1 and Clp. [file MPP-20-51-s006.docx]

**Supplementary Table S3.** Genes regulated by both the globe transcriptional regulator HpaR1 and Clp.

| Functional category | ORF number in *Xcc* strain 8004 (or ATCC33913) | Gene name | Predicted product | Expression fold change (*hpaR1*-/wt) | Expression fold change (*clp-*/wt) (From He *et al*., 2007) |
| --- | --- | --- | --- | --- | --- |
| Amino acids biosynthesis |  |  |  |  |  |
| Biosynthesis of cofactors, prosthetic groups, carriers |  |  |  |  |  |
| Cell envelope and cell structure | *XC_1459* (*XCC2658*) | *phuR* | outer membrane hemin receptor | 2.11 | 4.1 |
| Cellular processes | *XC_1410* (*XCC2704*)  *XC_2234* (*XCC1952*)  *XC_2237 (XCC1949*)  *XC_2238* (*XCC1948*)  *XC_2243* (*XCC1943*)  *XC_2245* (*XCC1941*)  *XC_2263* (*XCC1923*)  *XC_2267* (*XCC1919*)  *XC_2270* (*XCC1916*)  *XC_2277* (*XCC1910*)  *XC_2278* (*XCC1909*)  *XC_2280* (*XCC1907*) | *cheR*  *flgB*  *flgK*  *flgL*  *fliI*  *fliK*  *fliM*  *fliO*  *fliP*  *flhB*  *flhA*  *fleN* | response regulator for chemotaxis  flagellar basal body rod protein FlgB  flagellar hook-associated protein FlgK  flagellar hook-associated protein FlgL  flagellar protein  flagellar protein  flagellar motor switch protein FliM  flagellar protein  flagellar biosynthesis protein FliP  flagellar biosynthesis protein FlhB  flagellar biosynthesis protein FlhA  flagellar biosynthesis switch protein | 3.26  3.08  2.18  2.19  2.38  2.39  2.73  2.18  2.24  2.13  2.33  2.93 | -9.3  -3.8  -1.7  -2.8  -3.5  -2.4  -3.5  -3.8  -3.3  -3.1  -2.1  -3.3 |
| Central intermediary metabolism |  |  |  |  |  |
| Energy and carbon metabolism | *XC_0279* (*XCC0269*)  *XC_0281* (*XCC0271*)  *XC_3683* (*XCC0549*) | *mocA*  *atpE* | 2,5-diketo-D-gluconate reductase B  oxidoreductase  F0F1 ATP synthase subunit C | -2.63  -2.80  -2.56 | -4.1  -5.0  -12.3 |
| Fatty acid and phospholipid meatbolism |  |  |  |  |  |
| Nucleotides metabolism |  |  |  |  |  |
| Regulatory functions |  |  |  |  |  |
| Replication and DNA metabolism |  |  |  |  |  |
| Transport | *XC_1341* (*XCC2772*)  *XC_1113* (*XCC3045*)  *XC_2844* (*XCC1393*)  *XC_3201* (*XCC1045*)  *XC_3293* (*XCC0942*) | *fhuA*  *bfeA*  *brf*  *cysW* | TonB-dependent receptor  ferric enterobactin receptor  bacterioferritin  bacterioferritin  sulfate ABC transporter sulfate permease | 2.16  -2.15  -2.07  -4.16  -2.34 | -3.5  -4.7  -4.4  -4.7  -3.3 |
| Translation | *XC_0094* (*XCC0093*)  *XC_0096* (*XCC0094*)  *XC_0654* (*XCC3506*)  *XC_0667* (*XCC3494*)  *XC_1291* (*XCC2821*)  *XC_1292* (*XCC2820*) | *tldD*  *tldD*      *hslV* | TldD protein  TldD protein  prolyl oligopeptidase  ATP-dependent protease peptidase subunit  endoproteinase Arg-C  endoproteinase Arg-C | 2.32  2.53  2.37  -2.34  -2.72  -3.47 | -4.1  -3.3  -7.6  4.1  -7.1  -4.4 |
| Transcription |  |  |  |  |  |
| Signal transduction |  |  |  |  |  |
| Mobile genetic elements |  |  |  | \|  \| \| --- \| |  |
| Pathogenicity and adaptation | *XC_1811* (*XCC2304*)  *XC_3861* (*XCC3789*)  *XC_0639* (*XCC3521*)  *XC_0026* (*XCC0026*)  *XC_0027* (*XCC0027*)  *XC_1515* (*XCC2601*)  *XC_1664* (*XCC2448*)  *XC_1667* (*XCC2445*)  *XC_1668* (*XCC2444*)  *XC_1669* (*XCC2443*)  *XC_3590* (*XCC0645*)  *XC_3591* (*XCC0644*) | *acvB*  *acrA*  *engXCA*  *egl*  *egl*  *gumH*  *gumK*  *gumL*  *gumM*  *pel*  *pel* | virulence protein  acriflavin resistance protein  major extracellular endoglucanase  cellulase  cellulase  extracellular protease  GumH protein  GumK protein  GumL protein  GumM protein  pectate lyase  pectate lyase | 2.04  3.21  -3.04  -2.01  -4.53  -2.53  -2.06  -2.59  -2.98  -3.01  -2.67  -16.11 | 4.4  -6.6  -10.7  -3.5  -4.2  -4.1  -4.1  -3.8  -5.7  -4.7  -4.4  -4.4 |
| Undefined category |  |  |  |  |  |
| Hypothetical proteins | *XC_0657* (*XCC3504*)  *XC_1710* (*XCC2402*)  *XC_2740* (*XCC1469*) |  | hypothetical protein  hypothetical protein  hypothetical protein | 2.79  3.22  13.84 | -6.6  3.1  -3.3 |
