# Supplementary material for: Competitive control of endoglucanase gene engXCA expression in the plant pathogen Xanthomonas campestris by the global transcriptional regulators HpaR1 and Clp
Source: Mol Plant Pathol. 2018 Oct 9;20(1):51–68. doi: 10.1111/mpp.12739 (PMC6430473; doi:10.1111/mpp.12739)
Supplement: Supplementary file 7 — Table S4 Bacterial strains and plasmids used in this work. aRifr, Kanr, Tetr and Spcr indicate resistance to rifampicin, kanamycin, tetracycline and spectinomycin, respectively. [file MPP-20-51-s007.docx]

**Supplementary Table S4.** Strains and plasmids used in this study

| Strains or plasmids | Relevant characteristics | Reference or source |
| --- | --- | --- |
| *E. coli* |  |  |
| JM109 | *RecA*1*, endA*1*, gyrA*96*, thi, supE*44*, relA*1△ (*lac-proAB*)/F’ [*traD36, lacI*^q^, *lacZ* △M15] | (1) |
| DH5α | Φ80△*lacZM*15 *recA1 endA1 deoR* | Gibco BRL, Life Technologies |
| JM109/pQE-30-2736 | JM109 harbouring pQE-30-2736 | (2) |
| JM109/pQE-30-Clp | JM109 harbouring pQE-30-Clp | This work |
| *X. campestris* pv. *campestris* |  |  |
| 8004 | Wild type, Rif^r^ | (3) |
| 2736nk | As 8004, but *hpaR1*::pK18*mob*, non-polar effect. Rif^r^, Kan^r^ | (2) |
| ΔhpaR1 | As 8004, but *hpaR1* gene (*XC_2736*) deleted, non-polar effect. Rif^r^ | Author’s lab collection |
| CΔhpaR1 | ΔhpaR1 harboring a recombinant plasmid derived from the full length of *hpaR1* cloned into the promoterless plasmid pLAFR6. | Author’s lab collection |
| Δclp | As 8004, but *clp* gene (*XC_0486*) deleted, non-polar effect. Rif^r^ | Author’s lab collection |
| CΔclp | Δclp harboring a recombinant plasmid derived from the full length of *clp* cloned into the promoterless plasmid pLAFR6. | Author’s lab collection |
| ΔhpaR1/clp | As 8004, but both *hpaR1* and clp genes deleted, non-polar effect. Rif^r^ | Author’s lab collection |
| 8004/pGUSengXCA | 8004 harbouring pGUSengXCA, Rif^r^ Tet^r^ | This work |
| 8004/pGUSengXCA_AT_ | 8004 harbouring pGUSengXCA_AT_, Rif^r^ Tet^r^ | This work |
| 8004/pGUSengXCA_CA_ | 8004 harbouring pGUSengXCA_CA_, Rif^r^ Tet^r^ | This work |
| ΔhpaR1/pGUSengXCA | ΔhpaR1 harbouring pGUSengXCA, Rif^r^ Tet^r^ | This work |
| Δclp/pGUSengXCA | Δclp harbouring pGUSengXCA, Rif^r^ Tet^r^ | This work |
| 8004/ EngXCA::6×His | As 8004, but chromosomally encoding EngXCA fused with 6×His peptide at the C-terminus of the EngXCA. Rif^r^ | This work |
| ΔhpaR1/EngXCA::6×His | As HpaR1 deletion mutant ΔhpaR1, but chromosomally encoding EngXCA fused with 6×His peptide at the C-terminus of the EngXCA. Rif^r^ | This work |
| Δclp/EngXCA::6×His | As *clp* deletion mutant Δclp, but chromosomally encoding EngXCA fused with 6×His peptide at the C-terminus of the EngXCA. Rif^r^ | This work |
| ΔhpaR1/clp/EngXCA::6×His | As *hpaR1*/*clp* double deletion mutant ΔhpaR1/clp, but chromosomally encoding EngXCA fused with 6×His peptide at the C-terminus of the EngXCA. Rif^r^ | This work |
| ΔHpaR1/pHpa-Flag | ΔhpaR1 harbouring recombinant plasmid pHpa-Flag, Rif^r^ Tet^r^ | This work |
| ΔClp/pClp-Flag | Δclp harbouring recombinant plasmid pClp-Flag, Rif^r^ Tet^r^ | This work |
| Plasmids |  |  |
| pLAFR3 | Broad host range cloning vector, Tet^r^ | (4) |
| pLAFR6 | A promoterless derivative of pLAFR3, Tet^r^ | (5) |
| pRK2073 | Helper plasmid, Tra+, Mob+, ColE1, Spc^r^. | (6) |
| pQE-30 | Expression vector, allowing the production of fusion proteins containing amino terminal 6×His-tagged sequences. Ampr | Qiagen, Germany |
| pQE-30-2736 | pQE-30 containing a 360-bp fragment of *hpaR1* gene coding region. | (2) |
| pQE-30-Clp | pQE-30 containing a 690-bp fragment of *clp* gene coding region. | This work |
| pL6*gus* | pLAFR6 containing a 1,832-bp *gusA* ORF (excluding ATG), Tet^r^ | (7) |
| pGUSengXCA | pLAFR6 containing 303-bp promoter region of the gene *engXCA* fused to the coding region of *gusA*, Tet^r^ | This work |
| pGUSengXCA_AT_ | pLAFR6 containing the mutant *engXCA* promoter with 2 nucleotides substitution in -10 elements, fused to the coding region of *gusA*, Tet^r^ | This work |
| pGUSengXCA_CA_ | pLAFR6 containing the mutant *engXCA* promoter with 2 nucleotides substitution in -35 elements, fused to the coding region of *gusA*, Tet^r^ | This work |
| pK18*mobsacB* | pUC18 derivative, *lacZα*, *sacB*, Kan^r^, *mob* site. Allelic exchange vector (Suicidal vector carrying *sacB* gene for mutagenesis). | (8) |
| pK18*mobengXCA*H6 | pK18*mobsacB* containing the encoding sequence of EngXCA with 6×His tag in its C-terminus, Tet^r^ | This work |
| pK_engXCA_ | The suicide plasmid pK18*mob* containing 303-bp *engXCA* promoter (spanning nucleotides -256 to +47 relative to TIS). Used for site-directed mutagenesis. | This work |
| pK_eng317_ | The suicide plasmid pK18*mob* containing 317-bp *engXCA* promoter (spanning nucleotides -191 to +126 relative to the TIS). Used for site-directed mutagenesis. | This work |
| pHpa-Flag | pLAFR3 containing the encoding sequence of HpaR1 fused with 3×Flag-tag at the N-terminus of HpaR1. Tet^r^ | This work |
| pClp-Flag | pLAFR3 containing the encoding sequence of Clp fused with 3×Flag-tag at the N-terminus of Clp. Tet^r^ | This work |

Note: ^a^Rif^r^, Kan^r^, Tet^r^, and Spc^r^ indicate resistance to rifampicin, kanamycin, tetracycline, and spectinomycin, respectively.

**References**

1. Yanisch-Perron, C., Vieira, J. & Messing, J. (1985) Improved M13 phage cloning vectors and host strains: nucleotide sequences of the M13mp18 and pUC19 vectors. *Gene* **33**, 103–119.

2. An, S.Q., Lu, G.T., Su, H.Z., Li, R.F., He, Y.Q., Jiang, B.L., Tang, D.J. and Tang, J.L. (2011) Systematic mutagenesis of all predicted *gntR* genes in *Xanthomonas campestris* pv. *campestris* reveals a GntR family transcriptional regulator controlling hypersensitive response and virulence. *Mol. Plant Microbe Interact.*, **24**, 1027−1039.

3. Daniels, M.J., Barber, C.E., Turner, P.C., Sawczyc, M.K., Byrde, R.J. and Fielding, A.H. (1984) Cloning of genes involved in pathogenicity of Xanthomonas campestris pv. campestris using the broad host range cosmid pLAFR1. EMBO J., 3, 3323−3328.

4.Staskawicz, B., Dahlbeck, D., Keen, N., and Napoli, C. (1987) Molecular characterization of cloned avirulence genes fromrace 0 and race 1 of Pseudomonas *syringaepv.glycinea*. *J Bacteriol.* **169**, 5789–5794.

5. Huynh, T. V., Dahlbeck, D. & Staskawicz, B. J. (1989) Bacterial blight of soybean: regulation of a pathogen gene determining host cultivar specificity. *Science* **245**, 1374–1377.

6. Leong, S. A., Ditta, G. S. & Helinski, D. R. (1982) Heme biosynthesis in *Rhizobium*. Identification of a cloned gene coding for delta-aminolevulinic acid synthetase from *Rhizobium meliloti*. *J. Biol. Chem.* **257**, 8724–8730.

7. Jiang, B. L. *et al.* (2008) The type III secretion effector XopXccN of *Xanthomonas campestris* pv. *campestris* is required for full virulence. *Res. Microbiol.* **159**, 216–220.

8. Schäfer, A., Tauch, A., Jäger, W., Kalinowski, J., Thierbach, G. and Pühler, A. (1994) Small mobilizable multi-purpose cloning vectors derived from the *Escherichia coli* plasmids pK18 and pK19: selection of defined deletions in the chromosome of *Corynebacterium glutamicum*. *Gene*., **145**, 69−73.
