# Supplementary material for: Competitive control of endoglucanase gene engXCA expression in the plant pathogen Xanthomonas campestris by the global transcriptional regulators HpaR1 and Clp
Source: Mol Plant Pathol. 2018 Oct 9;20(1):51–68. doi: 10.1111/mpp.12739 (PMC6430473; doi:10.1111/mpp.12739)
Supplement: Supplementary file 8 — Table S 5 Primers used in this study. [file MPP-20-51-s008.docx]

**Supplementary Table S5.** Primers used in this study^§^

| Primer | Nucleotide sequence (5′→3′) | The amplified segment or the primer location |
| --- | --- | --- |
| O_engXCA_-F  O_engXCA_-R | ACAGTTGGATCC TCAGCACCAAGGTGATCGTG  ACAGTTAAGCTTTCAGTGATGGTGATGGTGATG GCTCGCGGCGCAGAAGCCGAAC | DNA fragment encoding EngXCA protein fused with 6×His-tag, used for constructing *Xcc* strains chromosomally encoding EngXCA::6×His protein. |
| P_engXCA_-1F  P_engXCA_-1R | ACAGTTGAATTC CGCCATTGCGCCAGTTCGGT  ACAGTTGGATCC CATGGTGATCTCCCTAGAGA | 303-bp DNA sequence upstream of the start codon of *engXCA* (include ATG, spanning nucleotides -256 to +47 relative to the TIS), used for constructing the reporter plasmid and plasmid for creating mutation. |
| P_engXCA_-2F  P_engXCA_-2R | ACAGTTGAATTC AGTTCGGTGCGCAGGAAGGA  ACAGTTGGATCC TGGAATAGCTGAAGGCCGGC | 317-bp DNA fragments spanning nucleotides -191 to +126 relative to the TIS of the *engXCA* promoter of *Xcc*, used for creating nucleotides substitution or deletion for *in vitro* transcription assay. |
| eng-ivtF  eng-ivtR | AGTTCGGTGCGCAGGAAGGA  TGGAATAGCTGAAGGCCGGC | 317-bp DNA fragments spanning nucleotides -191 to +126 relative to the TIS of the *engXCA* promoter of *Xcc*, used for *in vitro* transcription assay, or EMSA. |
| ck-ivtF  ck-ivtR | TTACCGCGCTCGGGTGTCGA  TGGAATAGCTGAAGGCCGGC | 126-bp DNA fragments spanning nucleotides +1 to +126 relative to the TIS of the *engXCA* promoter of *Xcc*, used as a control in *in vitro* transcription assay. |
| Dye-1F  Dye-1R | CGAACGTGGCAACGGCGCCAGCCATCAG  ACGTTCACACCCTTGAGCTGCACGACCTT | 440-bp DNA fragments spanning nucleotides -250 to +190 relative to TIS of the *engXCA* promoter of *Xcc*, used for dye primer-based DNase I footprint assay to determine HpaR1 binding site, FAM-labelled. |
| Dye-2F  Dye-2R | AGTTCGGTGCGCAGGAAGGA  TTCACACCCTTGAGCTGCACG | 378-bp DNA fragments spanning nucleotides -191 to +187 relative to the TIS of the *engXCA* promoter of *Xcc*, used for dye primer-based DNase I footprint assay to determined Clp binding site, FAM-labelled. |
| Dye-3F  Dye-3R | AGGCGATGACTTCGGGAATC  CCTGAATATGGACATGGTGATCTC | 333-bp DNA fragments spanning nucleotides -274 to +59 relative to the TIS of the *engXCA* promoter of *Xcc*, used for dye primer-based DNase I footprint assay to determined Clp binding site, FAM-labelled. |
| P_−256/+47_-F  P_−256/+47_-R | CGCCATTGCGCCAGTTCGGT  CATGGTGATCTCCCTAGAGAACCGGGC | 303-bp DNA fragments spanning nucleotides -256 to +47 relative to the TIS of the *engXCA* promoter of *Xcc*, used for EMSA, FAM-labelled. |
| P_−184/+47_-F  P_−184/+47_-R | TGCGCAGGAAGGACATGGGG  CATGGTGATCTCCCTAGAGAACCGGGC | 231-bp DNA fragments spanning nucleotides -184 to +47 relative to the TIS of the *engXCA* promoter of *Xcc*, used for EMSA, FAM-labelled. |
| P_−141/+47_-F  P_−141/+47_-R | AGTCCTGCGGACAGCGCGCA  CATGGTGATCTCCCTAGAGAACCGGGC | 188-bp DNA fragments spanning nucleotides -141 to +47 relative to the TIS of the *engXCA* promoter of *Xcc*, used for EMSA, FAM-labelled. |
| P_−91/+47_-F  P_−91/+47_-R | TCCCGCAGCCGCGATGTGAT  CATGGTGATCTCCCTAGAGAACCGGGC | 138-bp DNA fragments spanning nucleotides -91 to +47 relative to the TIS of the *engXCA* promoter of *Xcc*, used for EMSA, FAM-labelled. |
| P_−56/+47_-F  P_−56/+47_-R | GTTTTCTGTGGGGACGATCACACCA  CATGGTGATCTCCCTAGAGAACCGGGC | 103-bp DNA fragments spanning nucleotides -56 to +47 relative to the TIS of the *engXCA* promoter of *Xcc*, used for EMSA, FAM-labelled. |
| P_−36/+120_-F  P_−36/+120_-R | CACCACGCGACGCGCGCACA  AGCTGAAGGCCGGCCCGGCG | 156-bp DNA fragments spanning nucleotides -36 to +120 relative to the TIS of the *engXCA* promoter of *Xcc*, used for EMSA, FAM-labelled. |
| P_−64/+120_-F  P_−64/+120_-R | GCAATGGTGTTTTCTGTGGGG  AGCTGAAGGCCGGCCCGGCG | 184-bp DNA fragments spanning nucleotides -64 to +120 relative to the TIS of the *engXCA* promoter of *Xcc*, used for EMSA, FAM-labelled. |
| engRTR1  engRTR2  engRTR3 | GCATGGTGTCGCTACGCA  TCATGTCCTTCCAGTTGCGT  CGAACACGTTCACACCCTTG | Located in *engXCA* ORF, used for 5′-RACE |
| HBS-F  HBS-R | GGTGCGGCAATGGTACGATCACACCACGC  GCGTGGTGTGATCGTACCATTGCCGCACC | Used for creating 13 nucleotides (GTTTTCTGTGGGG) deletion within HBS on *engXCA* promoter via site-directed mutagenesis. |
| CBS I-F  CBS I-R | TCCCGCAGCCGCGAGCGGCAATGGTGTTT  AAACACCATTGCCGCTCGCGGCTGCGGGA | Used for creating 10 nucleotides deletion (TGTGATCGGT) within CBS I on *engXCA* promoter via site-directed mutagenesis. |
| AT10-F  AT10-R | CGCGCACAGACCAAGCGGCCCGCCTTACCGCG  cgcggtaaggcgggccgcttggtctgtgcgcg | Used for creating nucleotides substitution in -10 element of *engXCA* promoter via site-directed mutagenesis. |
| CA35-F  CA35-R | CTGTGGGGACGATCAGTCCACGCGACGCGCGCA  tgcgcgcgtcgcgtggactgatcgtccccacag | Used for creating nucleotides substitution in -35 element of *engXCA* promoter via site-directed mutagenesis. |
| 3077F  3077R | TCTCACTCTGTCTTGCAAACTGCGA  AATGGGCATCGAAAACCAGAAGC | 890-bp DNA fragment including the promoter region and coding region of *hrpG* of *Xcc* strain 8004, used as a control in *in vitro* transcription assay. |
| Clp-OF  Clp-OR | ACAGTTGGATCCATGAGCCTAGGGAACACGAC  ACAGTTAAGCTTTTAGCGCGTGCCGTACAACA | 693-bp DNA fragment of the *clp* ORF sequence, used for overproducing Clp protein. |
| Hflag-F  Hflag-R | CGGGATCCGACTACAAAGACCATGACGGTGATTATAAAGATCATGATATCGACTACAAAGATGACGACGATAAAATGACTGACATCCAGTGGAGC  CCAAGCTTTCATGGTGTTTTCCCCTGTG | DNA fragment encoding HpaR1 fused with 3×Flag-tag N-terminally, used for constructing *Xcc* strain producing 3×Flag::HpaR1 protein. |
| Cflag-F  Cflag-R | CGGGATCCGACTACAAAGACCATGACGGTGATTATAAAGATCATGATATCGACTACAAAGATGACGACGATAAAATGAGCCTAGGGAACACGACG  CCAAGCTTTTAGCGCGTGCCGTACAACA | DNA fragment encoding Clp protein fused with 3×Flag-tag N-terminally, used for constructing *Xcc* strain producing 3×Flag::Clp protein. |
| 0639F 0639R | GACAAGAACGACAGTCCTGCGG  AGGCGGGCATCTTGGTCTGT | DNA fragment of *engXCA* promoter, used for ChIP-PCR assay. |
| 0784F 0784R | CCAAGTAACCGCGGCGATCA  TCGCGCTCAGCACCAGATGT | DNA fragment of *XC_0784* promoter, used for ChIP-PCR assay. |
| gumB-F gumB-R | CGTTCTCTTAACGTCGAGGCGAC  AGCATTCAACCGGCTCAGGC | DNA fragment of *gumB* promoter, used for ChIP-PCR assay. |
| engXCA-F  engXCA-R | TCTGGTACACCGGCTCCTAT  CAGCCTTGTTCCAATCGGTG | 168-bp DNA fragment of the *engXCA* sequence, used for qRT-PCR. |
| 16SF  16SR | GCCTAACACATGCAAGTCGAACGGC  AATATTCCCCACTGCTGCCTCCCG | 325-bp DNA fragment of the 16S rDNA sequence, used for qRT-PCR. |
| 0251-F  0251-R | TTGTGTCAGCTCACTGGCG  CGAAAGCAGTCACGGAAGGT | 155-bp internal DNA fragment of the *XC_0251*, used for RT-PCR. |
| 0286-F  0286-R | ATCGGCGAGCGAAGAGGTCA  TGCGCATCCGACAGGGTCTT | 177-bp internal DNA fragment of the *XC_0286*, used for RT-PCR. |
| 0420-F  0420-R | AGAACAGCCCGAACACCCCA  ATGTGCAGCCAATCCAGCGC | 136-bp internal DNA fragment of the *XC_0420*, used for RT-PCR. |
| 0580-F  0580-R | GCCTGTGGGCCCATGAAAAG  CAGCCAGTTGTCGTCGGTCT | 161-bp internal DNA fragment of the *XC_0580*, used for RT-PCR. |
| 0992-F  0992-R | AGCGTCTGGTCGACGGTCTGGATGC  CATTGCCGGTCTGGCTGCCGTACAA | 177-bp internal DNA fragment of the *XC_0992*, used for RT-PCR. |
| 1005-F  1005R | CGCTCAAGGCGAAAATGGGTG  TGGCGGTGATGGGTGTGTTG | 165-bp internal DNA fragment of the *XC_1005*, used for RT-PCR. |
| 1087-F  1087-R | CCACAAGGAACTGCATGTCCGTGCG  GATCACCACGATGCGGTCAGCCAGT | 177-bp internal DNA fragment of the *XC_1087*, used for RT-PCR. |
| 1292-F  1292-R | GCGTTGTATCTCGCGTTGTTCTCC  AATTGCGCAACACGGGTTGG | 173-bp internal DNA fragment of the *XC_1292*, used for RT-PCR. |
| 1408-F  1408-R | ACGCAAGATCTTCAAGTGGGCGG  TGGTCGATTGGCGTTGCCTG | 153-bp internal DNA fragment of the *XC_1408*, used for RT-PCR. |
| 1544-F  1544-R | TTGACTGTTGCTCCTGGAGC  TTGCTTTGGTCCAGGGTCAG | 179-bp internal DNA fragment of the *XC_1544*, used for RT-PCR. |
| 1658-F  1658-R | CTGGCAATGTCCACGGTGCA  TTTCCAGTTCGCCAACGCCC | 178-bp internal DNA fragment of the *XC_1658*, used for RT-PCR. |
| 1972-F  1972-R | TTCCTGTTCGTGCAGTTGCCGTACA  AGAACTCGATGAAGATGTTGGCGCC | 157-bp internal DNA fragment of the *XC_1972*, used for RT-PCR. |
| 2235-F  2235-R | GACCTCGGTGAACGTCAAAG  AGTTACGCGAGGCGGAAATC | 158-bp internal DNA fragment of the *XC_2235*, used for RT-PCR. |
| 2239-F  2239-R | GACCGACTTCATCAACCCCT  CACCAGCTCTTCCACCGTAT | 169-bp internal DNA fragment of the *XC_2239*, used for RT-PCR. |
| 2277-F  2277-R | GAGCAAGGCAACATCCCGCAATCGC  AGTTGCAGCAGCAGGTCGCCGAAGT | 200-bp internal DNA fragment of the *XC_2277*, used for RT-PCR. |
| 3117-F  3117-R | ATGATCCGTGTGTGCCTGGT  CATGTCCATCAGCACCACGT | 165-bp internal DNA fragment of the *XC_3117*, used for RT-PCR. |
| 3128-F  3128-R | GAAGGAACAGTGGTTCACCAGCCAG  ACGACTTCAGCAGCGACACCAGCGT | 200-bp internal DNA fragment of the *XC_3128*, used for RT–PCR. |
| 3201-F  3201-R | ATGTCTACACAGCCAGCCAAC  CACACGTATTCGGTGGCAAG | 170-bp internal DNA fragment of the *XC_3201*, used for RT-PCR. |
| 3456-F  3456-R | ACCACGTGCTGGTGTTCCGCGATCA  CAGTAATGGCCGGCATCACCGAGCC | 190-bp internal DNA fragment of the *XC_3456*, used for RT-PCR. |
| 4010-F  4010-R | GGCATGACAGAACCCGTCGA  GTCAACGCCGCCAACATGTG | 158-bp internal DNA fragment of the *XC_4010*, used for RT-PCR. |

^§^The underlined sequences indicate the restriction sites for *Bam*HI, *Eco*RI and *Hin*dIII, respectively.
